# Boosting High-Voltage Practical Lithium Metal Batteries with Tailored Additives

**DOI:** 10.1007/s40820-024-01479-1

**Published:** 2024-07-29

**Authors:** Jinhai You, Qiong Wang, Runhong Wei, Li Deng, Yiyang Hu, Li Niu, Jingkai Wang, Xiaomei Zheng, Junwei Li, Yao Zhou, Jun-Tao Li

**Affiliations:** 1 College of Energy, Xiamen University, Xiamen, 361005 People’s Republic of China; 2 Magnetism Key Laboratory of Zhejiang Province, College of Materials and Chemistry, China Jiliang University, Hangzhou, 310018 People’s Republic of China; 3 Laboratory for Soft Matter and Biophysics, Department of Physics and Astronomy, KU Leuven, 3001 Leuven, Belgium; 4 State Key Lab of Physical Chemistry of Solid Surface, College of Chemistry and Chemical Engineering, Xiamen University, Xiamen, 361005 People’s Republic of China; 5 Department of Chemical Engineering, KU Leuven, Celestijnenlaan 200F, 3001 Leuven, Belgium; 6 College of Chemistry and Chemical Engineering, Northwest Normal University, Lanzhou, 730070 Gansu People’s Republic of China

**Keywords:** Li metal anode, Li dendrites, LiNO_3_, 1,3,6-tricyanohexane, Pouch cells

## Abstract

The lithium (Li) metal anode is widely regarded as an ideal anode material for high-energy-density batteries. However, uncontrolled Li dendrite growth often leads to unfavorable interfaces and low Coulombic efficiency (CE), limiting its broader application. Herein, an ether-based electrolyte (termed FGN-182) is formulated, exhibiting ultra-stable Li metal anodes through the incorporation of LiFSI and LiNO_3_ as dual salts. The synergistic effect of the dual salts facilitates the formation of a highly robust SEI film with fast Li^+^ transport kinetics. Notably, Li||Cu half cells exhibit an average CE reaching up to 99.56%. In particular, pouch cells equipped with high-loading lithium cobalt oxide (LCO, 3 mAh cm^−2^) cathodes, ultrathin Li chips (25 μm), and lean electrolytes (5 g Ah^−1^) demonstrate outstanding cycling performance, retaining 80% capacity after 125 cycles. To address the gas issue in the cathode under high voltage, cathode additives 1,3,6-tricyanohexane is incorporated with FGN-182; the resulting high-voltage LCO||Li (4.4 V) pouch cells can cycle steadily over 93 cycles. This study demonstrates that, even with the use of ether-based electrolytes, it is possible to simultaneously achieve significant improvements in both high Li utilization and electrolyte tolerance to high voltage by exploring appropriate functional additives for both the cathode and anode.

**Supplementary Information:**

The online version contains supplementary material available at 10.1007/s40820-024-01479-1.

## Highlights

- FGN-182 electrolytes exhibit highly reversible Li plating/stripping with an average Coulombic efficiency reaching up to 99.56% determined from Auerbach’s test.
- The gas-evolution process of LiNO_3_ in high-voltage lithium cobalt oxide (LCO) cathodes is revealed by in situ differential electrochemical mass spectrometry.
- Pouch cells equipped with high-loading LCO (3 mAh cm^−2^) cathodes, ultrathin Li chips (25 μm), and lean electrolytes (5 g Ah^−1^) using optimized electrolyte (FGN-182 + 1%HTCN) demonstrate outstanding cycling performance.

**Supplementary Information:**

The online version contains supplementary material available at 10.1007/s40820-024-01479-1.

## Graphical abstract

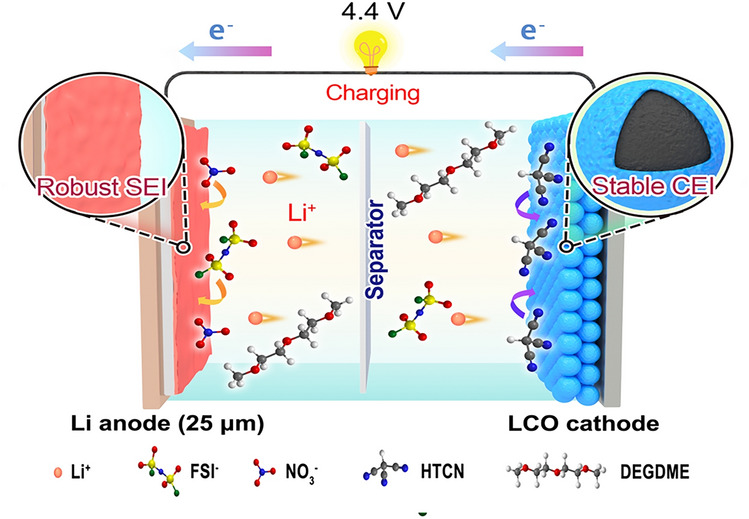

## Introduction

The ongoing development of electric vehicles and portable electronics underscores the urgent need for the next generation of efficient rechargeable batteries [1–3]. Notably, lithium (Li)-ion batteries have garnered considerable attention owing to their high energy density [4–7]. However, the conventional graphite anode, providing a capacity of only 372 mAh g^−1^, falls short of meeting the evolving demands of society. Li metal is widely recognized as an ideal anode material for Li metal batteries (LMBs) due to its exceptionally high theoretical specific capacity (3860 mAh g^−1^) and lowest reduction potential (− 3.04 V vs. SHE) [8–11]. Despite their broad application prospects, LMBs encounter several fundamental challenges and technical limitations. Of particular concern is the ever-growing Li dendrites and the occurrence of parasitic reactions with most electrolytes, which leads to poor cycling performance and safety concerns. Given that the formation of Li dendrites and interfacial reactions are heavily influenced by the surface characteristics of Li metal anodes, establishing an effective solid electrolyte interphase (SEI) on the Li surface emerges as a crucial approach for enhancing the performance of LMBs. Meanwhile, it is also crucial to consider the thickness of the Li metal anode, as practical applications necessitate energy density considerations. This oversight can lead to an excessive amount of Li metal anode in the evaluation of the full cell, making it difficult to accurately assess its true performance. Therefore, it is essential to use a limited amount of Li in full-cell testing to ensure a more realistic evaluation of overall battery performance.

Various strategies have been proposed to achieve stable Li metal anodes, including electrolyte optimization [12–16], artificial SEI layers [17–21], three-dimensional (3D) composite anodes [22–27], and solid-state electrolytes [28–33]. Among these approaches, the use of electrolyte additives stands out as a simple and effective method for controlling the nucleation and growth behavior of Li deposition and has been extensively investigated. In general, the Fermi level of Li metal is higher than the lowest unoccupied molecular orbital (LUMO) of almost all electrolytes (including solvents and Li salts), leading to the inevitable reduction of electrolyte molecules on the Li surface to form a SEI film. In addition, the resulting SEI film is typically nonuniform, exhibiting poor Li^+^ conductivity, and thereby leading to the growth of Li dendrites due to heterogeneous Li nucleation. Hence, compared to electrolyte molecules, the incorporation of desirable additives with lower LUMO energy levels can facilitate their earlier reduction on the Li surface, resulting in the formation of a robust SEI film with fast Li^+^ kinetics. This film, in turn, effectively governs the uniform Li deposition, thereby enabling the production of dendrite-free Li metal anodes. Wang et al. proposed using glycolide as an additive to enhance the cycling stability of ultrathin Li metal anodes, as it decomposes on the Li surface to enrich the organic components in the SEI, thereby improving the uniformity of Li deposition [34]. Qian and colleagues demonstrated the formation of dense SEI passivation films on the surface of Li metal anode, derived from the electrochemical decomposition of NFSALi attributed to its lower LUMO energy levels [35]. Hence, LiNi_0.5_Mn_1.5_O_4_||Li LMBs utilizing NFSA-containing electrolytes exhibited remarkable cycling stability, retaining 93% capacity after 400 cycles, along with improved Coulombic efficiency. Very recently, Li et al. introduced LiDFP and LiNO_3_ into the electrolyte to adjust the SEI composition, leveraging their lower LUMO energy levels compared to the solvents for preferential reducibility, resulting in a stable SEI containing LiF and Li_3_N, promoting rapid Li^+^ transport and uniform Li deposition [36].

While numerous electrolyte additives have been identified to enhance the stability of Li metal anodes, they may, however, have detrimental effects on the cathode, a factor that is often overlooked; in particular, such side effects can be amplified for high-voltage cathodes. For instance, desirable additives with relatively lower LUMO energy levels tend to preferentially reduce at the anode, leading to the formation of a stable SEI film. However, if these additives possess relatively high highest occupied molecular orbital (HOMO) energy levels, they may undergo oxidation at lower potentials before fully charged. Therefore, while additives with low LUMO energy levels may contribute to SEI stability, their compatibility with high-voltage positive electrodes must also be carefully considered in full cells. Although LiNO_3_ is well-known for its beneficial stabilizing effect on Li metal anodes, its influence on the cathode side, especially in high-voltage applications, has been given limited attention. Under high-voltage conditions, LiNO_3_ may undergo oxidation processes, potentially leading to the generation of gases or other unfavorable by-products. These reactions could adversely affect the performance and lifespan of the battery, highlighting the need for a comprehensive understanding and optimization of electrolyte components for enhanced battery cycling performance. Moreover, numerous reports indicate that ether-based electrolytes, compared to their carbonate-based counterpart, exhibit stronger compatibility with Li metal; however, suffering from their relatively poor oxidative stability (< 4 V), there are few reports of ether-based electrolyte that successfully balance both stable Li metal anodes and high-voltage (> 4.2 V) cathodes.

**Scheme 1:**
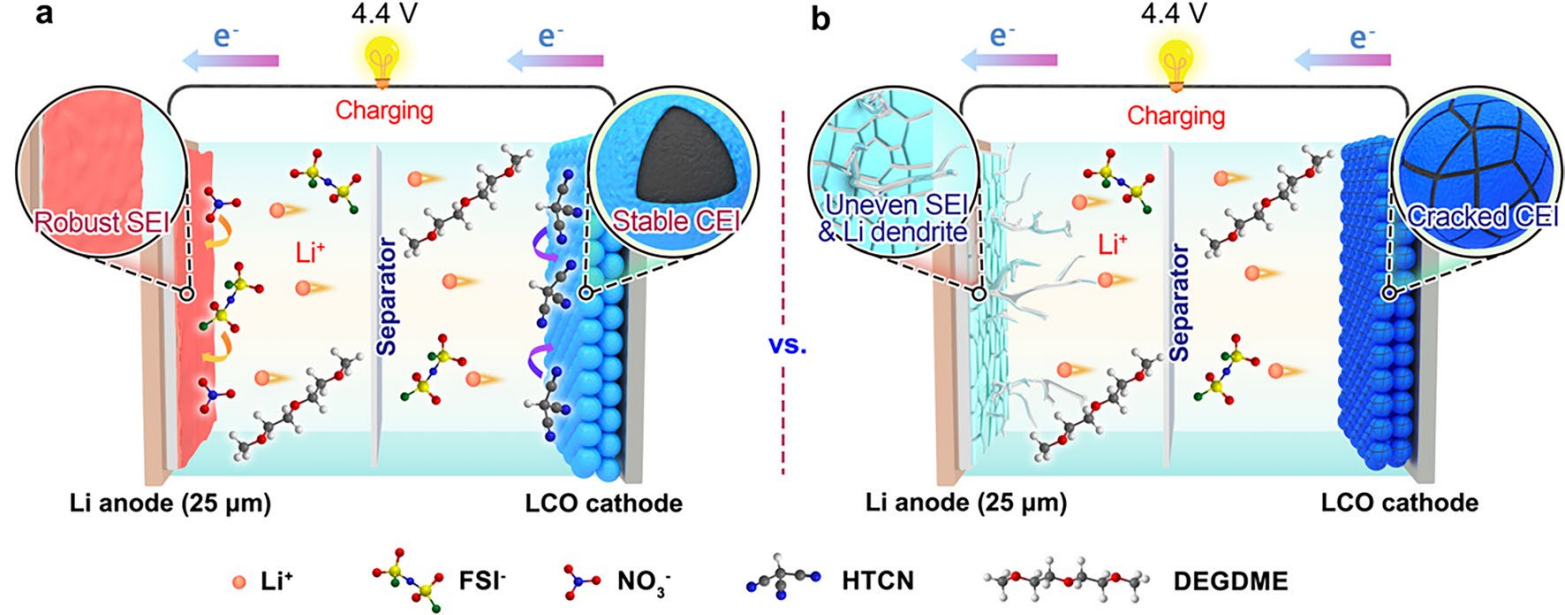
Schematic diagram illustrating high-voltage full cells utilizing **a** FGN-182 + 1%HTCN and **b** FGN-180 electrolytes, as well as their respective effects on the SEI for Li metal anodes and the CEI for LCO cathodes

In this study, an ether-based electrolyte (FGN-182) is developed that exhibits remarkable stability toward Li metal anodes by incorporating LiFSI and LiNO_3_ as dual salts. The synergistic effect of the dual salts facilitates the formation of a highly robust SEI film with desirable Li^+^ kinetics, thereby efficiently regulating Li deposition and preventing dendrite growth. Consequently, Li||Li symmetric cells utilizing FNG-182 electrolytes demonstrate outstanding stability in Li plating/stripping operations for over 1600 h, and Li||Cu half cells exhibit an average Coulombic efficiency (CE) reaching up to 99.56% as determined by the Auerbach test. The coin-cell configurations with high-loading LiNi_1/3_Co_1/3_Mn_1/3_O_2_ (NCM111, 2.5 mAh cm^−2^) and ultrathin Li chips (25 μm) result in a low negative/positive (N/P) ratio of 2.06, providing remarkable cycling stability with 80% capacity retention over 140 cycles at 0.5 C. Moreover, in order to investigate high-voltage applications (charged to 4.4 V), pouch cells are constructed using lithium cobalt oxide (LCO) electrodes loaded at 3 mAh cm^−2^, 25 μm Li foils (resulting in an N/P ratio of 1.7), and lean electrolytes at 5 g Ah^−1^. The results reveal that the presence of LiNO_3_ adversely affected the cathode performance at high voltage (gas generation), resulting in an 80% capacity retention after only 28 cycles. To address this issue, the cathode additive 1,3,6-tricyanohexane (HTCN) is further introduced; in the ultimately optimized electrolyte (FGN-182 + 1%HTCN), stable cycling could be extended to 93 cycles. Therefore, considerations are made for both the cathode and anode in such a formula, effectively enhancing the cycling performance of full cells (depicted in Scheme 1a), and thereby providing valuable insights for the practical application of high-voltage LMBs. However, in the baseline electrolyte (FGN-180), not only does the Li metal anode suffer ongoing Li dendrite growth due to an uneven SEI film, but also the cathode electrode fails to form a stable CEI film, leading to rapid material degradation, as illustrated in Scheme 1b.

## Results and Discussion

### Physicochemical Characterization of Electrolytes

Considering the excellent solubility of LiNO_3_, ether solvents, specifically, two commonly used ether solvents, 1,2-dimethoxyethane (DME) and diethylene glycol dimethyl ether (DEGDME), were employed as the electrolyte base in this study. Their HOMO energy levels are depicted in Fig. S1. While DME is a conventional electrolyte solvent in Li–S batteries [37, 38], it possesses a relatively narrow electrochemical window. As shown in the Fig. S1b, DEGDME (− 5.6053 eV) exhibited a lower HOMO energy level compared to DME (− 5.2137 eV) due to its longer methylene ether chain, suggesting enhanced oxidative stability, which holds promise for application in high-voltage LMBs. Extending the length of the methylene ether chain, such as with triethylene glycol dimethyl ether and tetraethylene glycol dimethyl ether, may indeed improve oxidative stability. However, this study refrains from further exploration of this aspect due to the anticipated rise in viscosity associated with increased chain length.

To examine the influence of LiNO_3_, this study varied the LiFSI:DEGDME:LiNO_3_ ratio across different proportions: 1:8:0, 1:8:0.2, 1:8:0.4, 1:8:0.8, 1:8:1, 1:8:2, and 1:8:3 (referred to as FGN-180, FGN-180.2, FGN-180.4, FGN-180.8, FGN-181, FGN-182, and FGN-183, respectively). Here, the LiFSI:DEGDME ratio of 1:8 approximately corresponds to 1 M Li salt. The viscosity and conductivity of various electrolytes were initially investigated, and the findings are shown in Fig. S2. The conductivity of FGN-180 electrolytes was measured at 7.5 mS cm^−1^, which gradually decreases with the addition of LiNO_3_. Specifically, the conductivities were 7.3, 6.8, 6.1, 5.7, 3.9, and 2.6 mS cm^−1^ for FGN-180.2, FGN-180.4, FGN-180.8, FGN-181, FGN-182, and FGN-183 electrolytes, respectively (Fig. S2). The decrease in conductivity observed with increasing Li^+^ concentrations (> 1 mol L^−1^) is consistent with earlier research [39]. In contrast, viscosity exhibited the opposite pattern, rising from 10.3 mPa s for FGN-180 to 14.1 mPa s for FGN-182, and significantly further to 26.3 mPa s for FGN-183 electrolytes. While LiNO_3_ is acknowledged for enhancing Li metal anode performance, the substantial increase in viscosity linked to higher LiNO_3_ concentrations suggests a trade-off.

### Electrochemical Performance of Li||Cu and Li||Li Symmetrical Cells

**Fig. 1:**
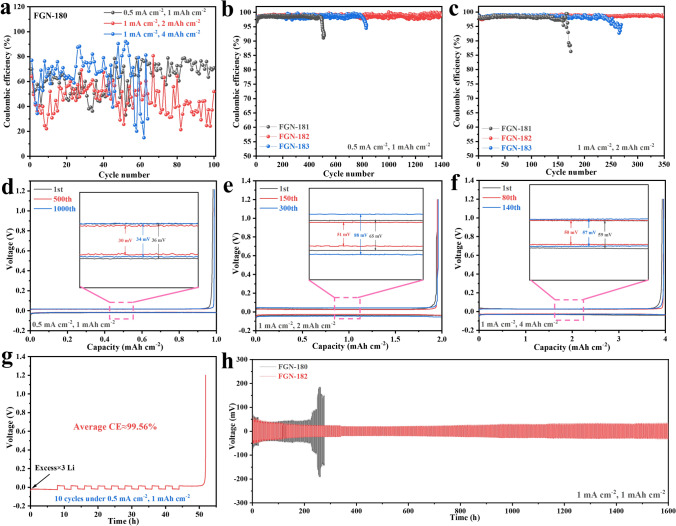
CE of Li||Cu cells using **a** FGN-180 and **b, c** FGN-181, FGN-182, and FGN-183 electrolytes at different currents/capacities. **d**–**f** Voltage profiles of FGN-182 electrolytes at different cycle numbers. **g** CE of Li plating/stripping in FGN-182 electrolytes using Auerbach’s method. **h** Cycling performance of the Li||Li symmetric cells using FGN-180 and FGN-182 electrolytes

The CE of Li plating/stripping on Li||Cu coin cells was assessed using various electrolytes under different conditions (Figs. 1a–c and S3). At 0.5 mA cm^−2^ with a capacity of 1 mAh cm^−2^, the CE of the FGN-180 electrolyte is notably low (approximately 70%, cf. Fig. 1a), significantly inferior to traditional DME-based electrolytes (> 95%). This could be attributed to its failure to form a stable SEI film. In contrast, in electrolytes with a low LiNO_3_ content (FGN-180.2 electrolytes, cf. Fig. S3), the CE significantly increases to 95% after 20 cycles. This improvement is attributed to the formation of a robust SEI film, although the CE begins to decline after 100 cycles. As anticipated, the CE substantially improves with LiNO_3_ addition. For instance, in the FGN-181 electrolyte, the CE remained above 98% for over 400 cycles (Fig. 1b). Remarkably, the FGN-182 electrolyte achieved a CE of around 99% even after 1400 cycles, with an average CE of 98.82% between the 1200th and 1400th cycles (Fig. S4a). Additionally, Fig. 1d illustrates the capacity-voltage profiles for the Li plating/stripping process of FGN-182 electrolytes across different cycles. As depicted by the corresponding polarization curves, the FGN-182 exhibited an overpotential of 36 mV during the 1st cycle, which decreased to 30 mV in the 500th cycle, and then slightly rose to 34 mV in the 1000th cycle, delivering a notable low Li plating/stripping overpotential during prolonged cycling. However, further increasing the LiNO_3_ content (FGN-183 electrolytes) yielded a stable CE for only 800 cycles before encountering a decline, likely attributable to a substantial increase in viscosity (cf. Fig. S2).

For comparison, bare LiNO_3_ in DEGDME with varying concentrations was also evaluated. As shown in Fig. S5**,** the electrolyte containing 1 M LiNO_3_ exhibited a CE of less than 95% and started to decline after 50 cycles. Despite the enhanced stability resulting from the addition of LiNO_3_, the CE only reached around 95% even in fully saturated LiNO_3_ electrolytes (between 5 and 7 M) and deteriorates to below 10% after 240 cycles. This implies that the presence of LiNO_3_ alone is inadequate, despite its ability to effectively regulate Li deposition. Further conductivity tests revealed that although the conductivity of the pure LiNO_3_ electrolyte (in DEGDME) increased with higher Li salt concentrations, it remained low, reaching only 0.69 mS cm^−1^ even in a saturated LiNO_3_ electrolyte (Fig. S6), which is less than one-tenth that of the FGN-180 electrolyte. This emphasizes the synergistic interaction between LiFSI and LiNO_3_ in forming a stable Li metal anode.

When the current density was enhanced to 1.0 mA cm^−2^ (with a capacity of 2 mAh cm^−2^), the CE of FGN-182 electrolytes remained 98.8% even after 350 cycles (Fig. 1c), showing stable cycling, with an average CE of 98.74% between 300th and 350th (Fig. S4b). In addition, Fig. 1e shows the overpotential of only 65, 51, and 88 mV for FGN-182 at 1st, 150th, and 300th, respectively, while the FGN-181 electrolyte displayed a CE of 98.4% at 100 cycles and droped to 86.3% at 174 cycles. Similarly, the FGN-183 electrolyte delivered a long-term cycling performance with high CE for the first 200 cycles, but then experienced a decline after 250 cycles. Remarkably, even at a higher capacity of 4 mAh cm^−2^ with a current density of 1 mA cm^−2^, the CE of FGN-182 electrolytes was still maintained at 99.1% after 180 cycles (Fig. S7). Moreover, the average CE between 300th and 350th still yielded 98.84% (Fig. S4c). However, FGN-181 and FGN-183 electrolytes exhibited inferior stability, with a decreased CE observed after 60 and 140 cycles, respectively. Figure 1f displays the overpotential variation for FGN-182 electrolytes, showing a similar trend compared to lower current densities/capacities (Fig. 1d, e). As expected, the FGN-180 electrolyte exhibited poor stability upon increasing densities/capacities (Fig. 1a).

We further evaluated the CE values of Li metal anodes using Auerbach’s method to demonstrate the superiority of the optimized electrolyte [40]. The FGN-182 electrolyte exhibited a CE as high as 99.56%, indicating an exceptional plating/stripping process **(**Fig. 1g**)**. In contrast, the CE in the conventional ether-based and carbonate-based electrolytes was only 96.69% and 82.26% (Fig. S8a, b), respectively. These results suggest that cells using FGN-182 electrolytes exhibit highly reversible electrode reaction kinetics. To further emphasize the superior CE of our optimized electrolyte FGN-182, we have included a performance comparison of electrolyte optimizations in Table S1.

To verify the excellent electrochemical stability of Li anode by using FGN-182 electrolytes, Li||Li symmetric cells were assembled, followed by galvanostatic discharge/charge tests of the cells with a Li cycling amount of 1 mAh cm^−2^ under 1 mA cm^−2^ (Fig. 1h). During the initial 5 cycles of the symmetric cells using FGN-182 electrolytes, an overpotential of about 55.1 mV can be observed (Fig. S9a), which then reduced to 25.8 mV after 200 h due to the stabilization at the beginning (Fig. S9b). Moreover, the overpotential remained at about 40 mV even after 1000 h of cycling (Fig. S9c). In contrast, the FGN-180 exhibited an initial overpotential of approximately 69.8 mV and experienced a rapid increase in polarization, leading to cell failure in less than 300 h of cycling. The results indicate that the FGN-182 electrolyte effectively regulates Li deposition, preventing dendrite formation and significantly extending cell lifespan.

Electrochemical impedance spectroscopy (EIS) was conducted to investigate the interfacial stability of cells with FGN-180 and FGN-182 electrolytes (Fig. S10). After the first cycle, the FGN-182 electrolyte exhibited slightly lower impedance than FGN-180, indicating a more stable electrode interface. Notably, the Li electrode in FGN-180 electrolyte showed a dramatic decrease in impedance after 10 cycles, followed by a further decrease after 20 cycles, due to the formation of Li dendrites in the early stages of battery cycling. However, after 50 cycles, the FGN-180 electrolyte exhibited a significant increase in impedance, which can be attributed to the continuous formation of the SEI layer due to Li dendrite growth during prolonged cycling. In contrast, the impedance of the FGN-182 electrolyte decreased initially after 10 cycles, rose slightly after 20 cycles, and then remained stable after 50 cycles. This behavior indicates the formation of a stable SEI layer, which is facilitated by the incorporation of LiNO_3_.

### Analysis of Solvation Structure in Different Electrolytes

**Fig. 2:**
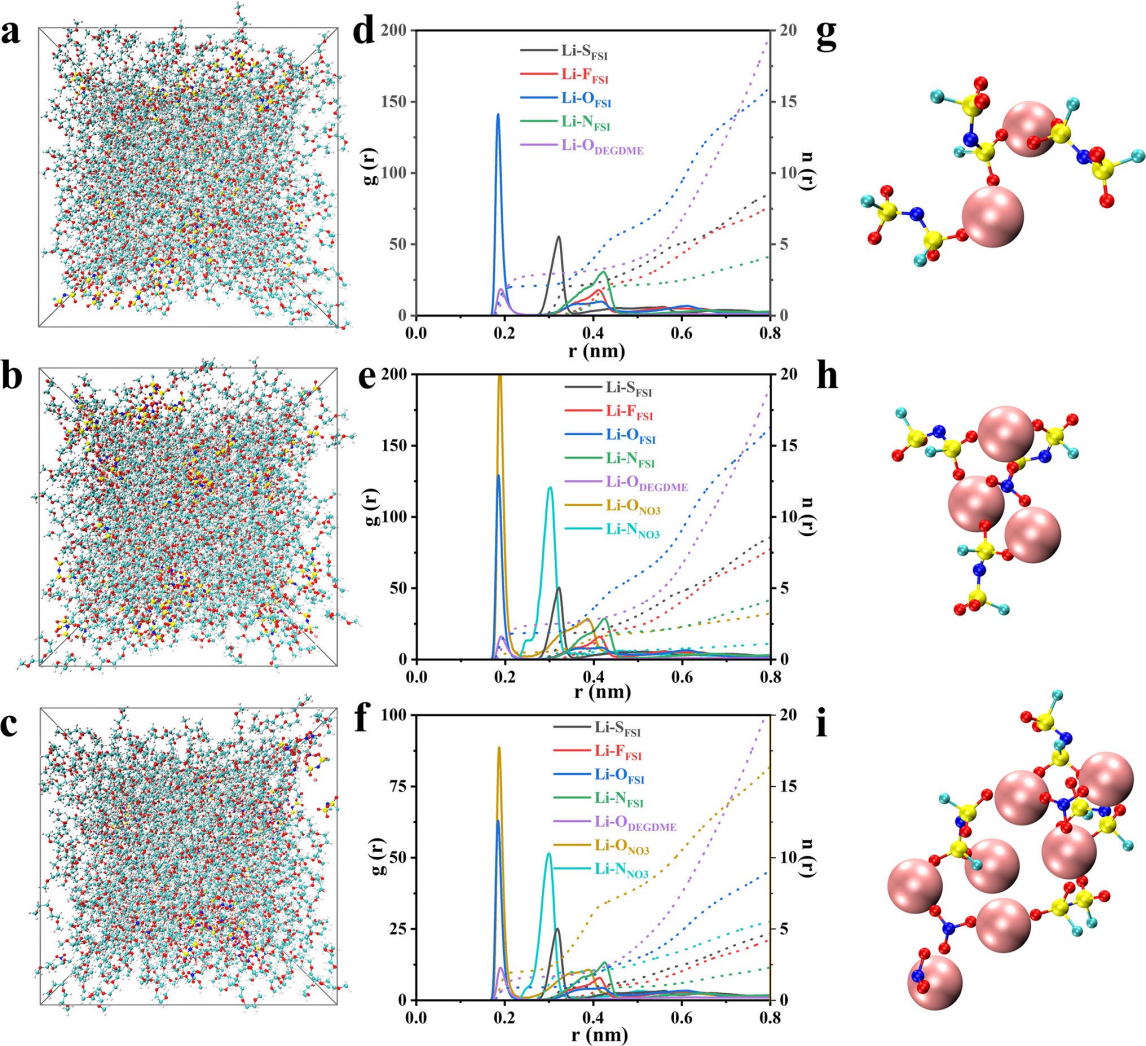
Snapshots of **a** FGN-180, **b** FGN-180.2, and** c** FGN-182 electrolytes obtained by MD simulation at 298 K. **d**–**f** Corresponding radial distribution functions (g (r), solid lines) and coordination numbers (n (r), dash lines) and **g-i** representative structural snapshots during MD simulation for each electrolyte. (Li, pink; N, blue; O, red; S, yellow; F, light blue). (Color figure online)

The solvation structure of FGN-180, FGN-180.2, and FGN-182 electrolytes was further investigated through theoretical simulations using molecular dynamics (MD). Figure 2a–c displays the relevant MD simulation snapshots. The initial configuration of each salt-solvent composite system was established by randomly distributing LiFSI, DEGDME, and LiNO_3_ molecules (Fig. 2g–i) according to their respective experimental molar ratios in the MD simulation box. According to the radial distribution function (RDF) (Fig. 2d–f**)** and the relevant coordination numbers (Tables S2 and S3**)**, the first Li^+^ coordination shell (within 1.90 Å) of FGN-180 primarily comprises Li–O_FSI_ and Li–O_DEGDME_, with coordination numbers of 2.071 and 2.778, respectively. This corresponds to the solvated FSI^−^ observed at Raman shifts of 720.1 and 1219.6 cm^−1^ [41], as well as the solvated DEGDME observed at 880.2 cm^−1^ (Fig. S11**,** red line) [42].

Following the addition of LiNO_3_, the RDF peaks of FGN-180.2 shifted to 1.84 Å for Li–O_FSI_ and 1.90 Å for Li–O_DEGDME_, with respective decreased coordination numbers of 1.852 and 2.370. Furthermore, a new peak emerged at 1.88 Å corresponding to Li–O_NO3_ with a coordination number of 0.496. These findings are corroborated by a new peak in the Raman shift at 1038.3 cm^−1^ (Fig. S11, blue line), indicating the incorporation of LiNO_3_ in the solvation structure of Li^+^. With a further increase of LiNO_3_ content, the coordination numbers corresponding to Li–O_FSI_ and Li–O_DEGDME_ of the FGN-182 decrease to 0.903 and 1.587, respectively. In contrast, the coordination numbers of Li–O_NO3_ increase to 2.037. These results imply the formation of a NO_3_^−^-rich solvation structure, which aligns with the Raman results revealing a prominent peak associated with NO_3_^−^, along with a slight blueshift (Fig. S11**,** green line). Therefore, the solvation structure analysis of FGN-182 electrolytes suggests its facilitative role in promoting the reduction of LiNO_3_ on the Li surface, thereby contributing to the formation of a stable SEI film.

### Characterization of Li Deposition Morphologies and Li^+^ Diffusion Kinetics in SEI

**Fig. 3:**
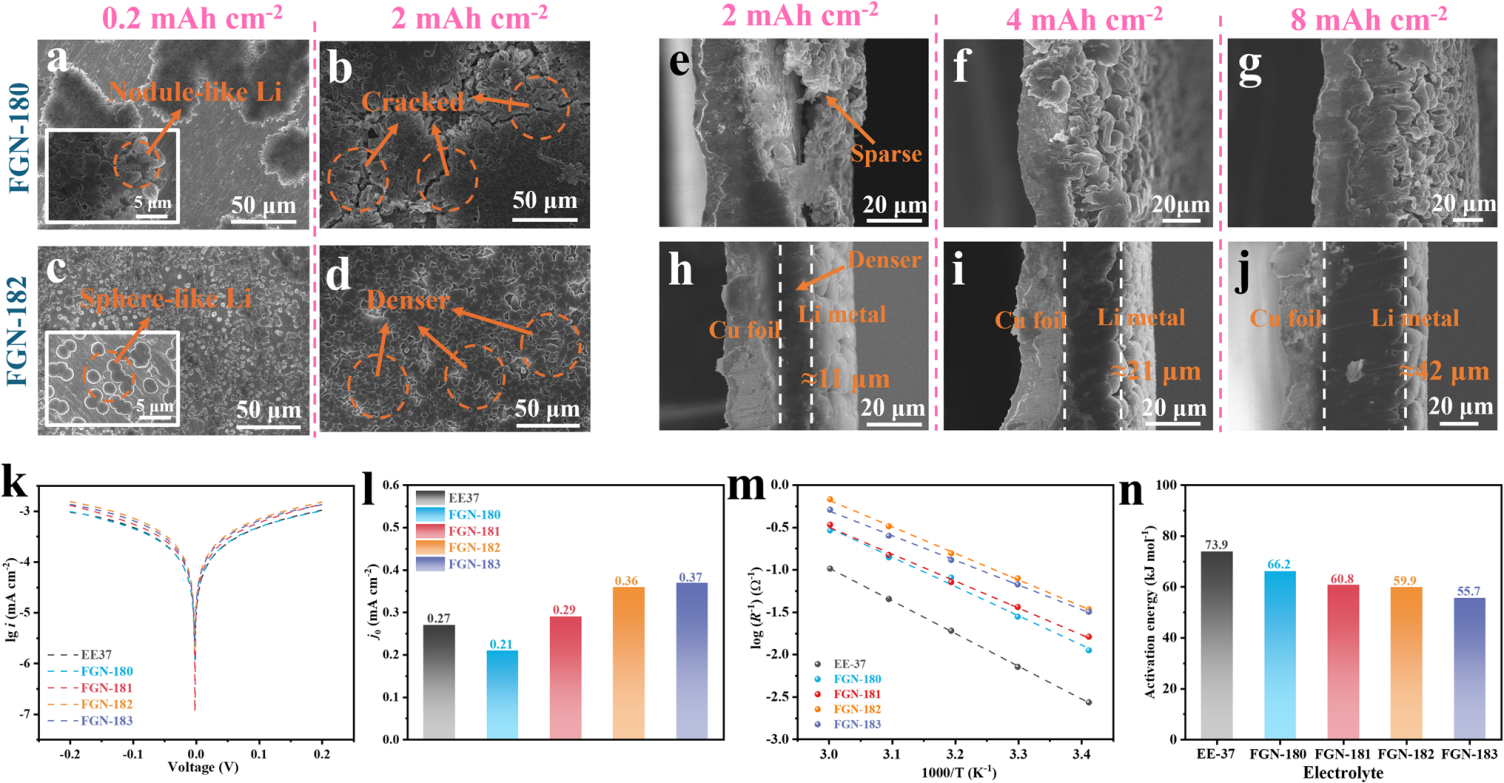
SEM images of Li metal deposited onto a Cu substrate on top view using **a, b** FGN-180 and **c, d** FGN-182 electrolytes, cross-sectional view for **e**–**g** FGN-180 and **h-j** FGN-182 electrolytes with different deposition capacities. **k** Tafel profiles and **l** exchange current density in different electrolytes. **m** Arrhenius plots for Li^+^ transport via the SEI in various electrolytes, and **n** the determined *E*_a_

The deposition behavior of Li metal onto Cu foil was then investigated using scanning electron microscopy (SEM). As shown in Fig. 3a, the FGN-180 electrolyte revealed uneven Li deposition, characterized by the growth of needle-like Li structures at a deposition amount of 0.2 mAh cm^−2^. Upon further increasing the deposition amount to 2 mAh cm^−2^ (Fig. 3b), cracks began to appear as a result of ongoing heterogeneous Li deposition.

However, with the addition of a small amount of LiNO_3_ (FGN-180.2 electrolytes (Fig. S12a), the deposition behavior of Li underwent a qualitative change, transitioning to the sphere-like Li, which is consistent with previous studies. At a deposition of 2 mAh cm^−2^, uniformly and compactly Li covered the entire Cu substrate** (**Fig. S12d). Furthermore, with a further increase in LiNO_3_ content, we found that the initial nucleation (0.2 mAh cm^−2^) tended to form smaller particles (cf. Figs. S12, S13, and 3c, d), and the sizes of Li particles were very similar in FGN-182 and FGN-183 electrolytes, indicating that further increasing the LiNO_3_ content had a negligible effect on the Li nucleation.

For comparison, bare LiNO_3_ dissolved in DEGDME was further studied; the sphere-like Li deposition (Fig. S14a–h) can be also observed at different concentrations of LiNO_3_ with a Li plating capacity of 0.2 mAh cm^−2^. As mentioned earlier, the size of Li particles decreased with increasing LiNO_3_ concentration from 1 to 3 M. However, with further increases in concentration (5 M and saturated), the reduction in Li particle size became negligible, indicating that particle size was no longer influenced by the LiNO_3_ concentration. Upon reaching a deposition capacity of 2 mAh cm^−2^, all electrolytes exhibited similar behavior, resembling the Li morphology observed in the FGN-182.

Considering that pure LiNO_3_ electrolytes (dissolved in DEGDME) only achieved around 95% CE, thus, the synergistic effect of LiFSI and LiNO_3_ is crucial for constructing a desirable SEI film thus obtaining higher CE. To further validate the superiority of electrolytes with optimized LiNO_3_ content, we also analyzed the cross-sectional SEM images of Li deposition morphologies in FGN-180 and FGN-182 electrolytes. As shown in Fig. 3e–g, the uneven Li deposition in FGN-180 electrolytes resulted in porous Li. Additionally, even at a deposition capacity of 8 mAh cm^−2^, needle-like Li formations were evident, corresponding to the previously noted low CE due to ineffective SEI film formation. A notable difference was observed in FGN-182 electrolytes, where the thicknesses at deposition capacities of 2, 4, and 8 mAh cm^−2^ yield 11, 21, and 42 μm, respectively (Fig. 3h–j). These values closely match the theoretical thickness of dense Li (1 mAh cm^−2^ ≈ 4.85 μm), further confirming the ultrahigh CE mentioned above.

The kinetics of Li migration beneath the SEI in different electrolytes was then assessed. The Tafel slopes of various electrolytes were investigated and extracted from the cyclic voltammetry (CV) curves depicted in Fig. 3k. Subsequently, the exchange current density (*j*_0_) was determined from the corresponding Tafel plots to characterize the charge-transfer kinetics within the SEI. As depicted in Fig. 3l, the carbonate-based electrolyte containing 1 M LiPF_6_ in EC/EMC (referred to as EE37) exhibited a slightly higher exchange current density of 0.27 mA cm^−2^ compared to that of FGN-180 electrolytes, which is only 0.21 mA cm^−2^. However, the incorporation of LiNO_3_ enhanced the exchange current density, reaching 0.29 and 0.36 mA cm^−2^ for FGN-181 and FGN-182 electrolytes, respectively, with a negligible improvement to 0.37 mA cm^−2^ for the FGN-183 electrolyte. This enhanced exchange current density further suggests accelerated SEI kinetics and, consequently, sufficient Li^+^ concentration availability beneath the SEI to facilitate spherical Li deposition, aligning with the earlier observations (Figs. 3a–d and S12-S13). In addition, the kinetics of Li^+^ transport of different SEI films generated in various electrolytes were further investigated using EIS of symmetric cells using Li electrodes across temperatures ranging from 20 to 60 °C (Fig. S15). The activation energy (*E*_a_) for Li^+^ transport via SEI films can be deduced utilizing the Arrhenius formula: $R_{{{\text{SEI}}}}^{ - 1} {\text{ = exp}}\left( { - \frac{{E_{{\text{a}}} }}{R \cdot T}} \right)$ [43, 44]. A lower *E*_a_ signifies greater dynamics of Li^+^ transport via the SEI layer. This leads to a higher concentration of Li^+^ beneath the SEI, facilitating the formation of larger Li particles rather than dendritic Li. As depicted in Fig. 3m, n, the *E*_a_ for Li^+^ diffusion through the SEI in the EE37 electrolyte was determined as 73.9 kJ mol^−1^, slightly reducing to 66.2 kJ mol^−1^ in the FGN-180 electrolyte, and further decreasing to 60.8 kJ mol^−1^ in the FGN-181 electrolyte. These findings suggest that the formation of a robust SEI film due to the synergistic effect of LiFSI and LiNO_3_ exhibits favorable Li^+^ transport characteristics. Consequently, smaller *E*_a_ values (59.9 and 55.7 kJ mol^−1^) were noted in FGN-182 and FGN-183 electrolytes, indicating an increased Li^+^ concentration beneath the SEI, thereby facilitating the formation of larger Li particles, consistent with the analysis of exchange current density and SEM results.

In addition, the Li^+^ transference number (*t*_Li+_) was assessed using EIS and chronoamperometry techniques for both FGN-180 and FGN-182 electrolytes. As illustrated in Fig. S16 and determined by the Bruce–Vincent equation (see Supporting Information), the *t*_Li+_ value for FGN-182 electrolytes is 0.63, which exceeds the value of 0.52 for FGN-180 electrolytes. According to Sand’s time formula, a higher *t*_Li+_ implies better suppression of Li dendrite formation. Therefore, it is anticipated that the FGN-182 electrolyte will effectively inhibit Li dendrite growth, thereby achieving uniform Li deposition.

### Characterization of SEI Composition

**Fig. 4:**
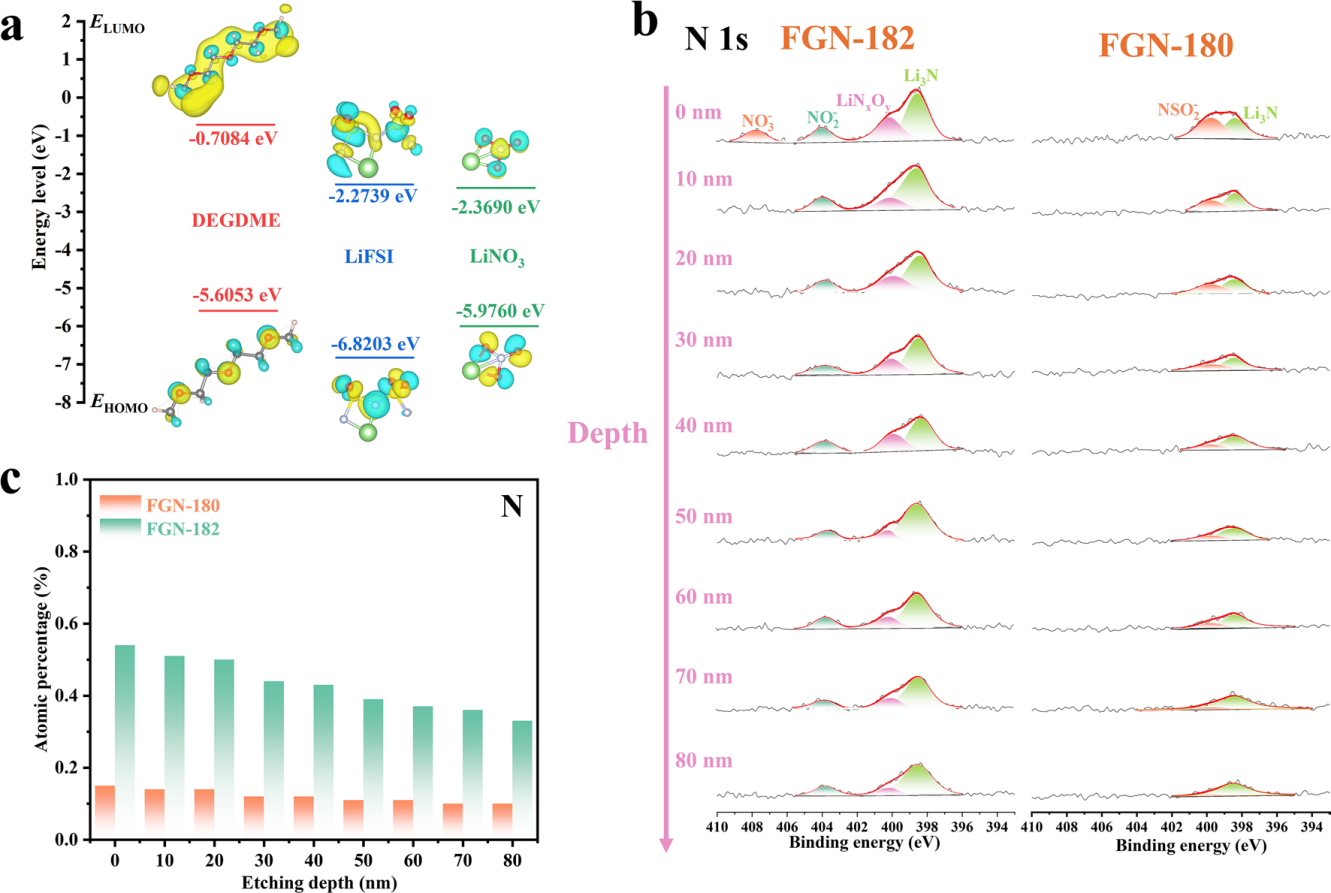
**a** HOMO and LUMO energies for DEGDME, LiFSI, and LiNO_3_. **b** In-depth XPS of N 1*s* spectra of SEI layer formed on cycled Li metal in FGN-182 and FGN-180 electrolytes. **c** Atomic percentage of N in SEI in FGN-182 and FGN-180 electrolytes with increasing etching depth

The composition of SEI is significantly impacted by the decomposition of solutes or solvents within the electrolyte. Utilizing density functional theory (DFT) based on the frontier molecular orbital theory, we calculated the molecular orbital energies of solvents and additives to elucidate their role in SEI formation. A lower LUMO energy level of the molecule suggests that its innermost unoccupied electron orbital is situated at a lower energy level, rendering it more prone to electron filling from external sources. Consequently, this facilitates the molecule’s reduction at higher voltages. As depicted in Fig. 4a, LiNO_3_ exhibited the lowest LUMO (− 2.3690 eV) energy level, indicating its priority in reduction and participate the formation of SEI film. This facilitated uniform Li^+^ transport with faster kinetics. In contrast to DEGDME (− 0.7084 eV), LiFSI (− 2.2739 eV) exhibited a lower LUMO energy level, indicating its preference for reduction to form the SEI film. However, despite its lower LUMO energy level, LiFSI alone demonstrates poor CE performance, suggesting its inadequacy in forming an effective SEI film when used as the sole component in the electrolyte. Similarly, electrolytes solely comprising LiNO_3_ also fail to achieve desirable CE, despite its recognized efficacy in enhancing Li deposition. Hence, the collaborative interplay of LiFSI and LiNO_3_ is crucial in the formation of the SEI film.

To investigate the composition and structure of the SEI film, in-depth X-ray photoelectron spectroscopy (XPS) analysis was further performed on the cycled Li electrode in FGN-180 and FGN-182 electrolytes. In the C 1*s* spectra (Fig. S17), the peaks at 284.8, 286.7, 288.8, and 289.9 eV correspond to C–C, C–O, C=O, and CO_3_^2−^ [45], respectively. These carbon peaks arise from the electrochemical reduction of DEGDME. The presence of an extremely weak signal at 290.6 eV for C–F suggests a possible unknown contaminant. The intensity of the C 1*s* spectrum exhibited slight changes with etching depth from the surface to 80 nm in both FGN-180 and FGN-182 electrolytes. Notably, as depicted in Fig. S20a, the proportion of C atoms in the SEI of the FGN-182 electrolyte consistently remained lower than that in FGN-180 with increasing etching depth. This suggests a decreased reduction of DEGDME molecules in the FGN-182 electrolyte, highlighting the formation of a stable SEI facilitated by the inclusion of LiNO_3_. Further examination of the O 1*s* spectrum revealed three distinct peaks at 530.9, 531.6, and 532.4 eV, corresponding to C=O, C–O, and CO_3_^2−^ [46], respectively (Fig. S18). In addition, the peak at 532.5 eV in the O 1*s* spectrum overlaps with the N–O bonding peak from LiNO_3_ in the FGN-182 electrolyte [47]. A low binding energy peak at approximately 528.2 eV, indicating Li_2_O [48], was observed with increasing etching depth for both electrolytes, suggesting that Li_2_O predominantly resides in the inner layer of the SEI film. Detailed analysis revealed a marginal difference in O content between FGN-180 and FGN-182 electrolytes (Fig. S20b). This difference arises from the higher decomposition of DEGDME contributing to SEI formation in the FGN-180 electrolyte compared to FGN-182, while LiNO_3_ serves as an additional O source in the FGN-182 electrolyte.

The F 1*s* spectra are depicted in Fig. S19 revealed negligible C–F signal on the surface of cycled Li in both electrolytes, with LiF being the predominant species. Moreover, with increasing etching depth, the LiF content in the FGN-182 gradually exceeds that in the FGN-180 electrolyte. As LiF is solely produced through the decomposition of LiFSI, these findings suggest that the inclusion of LiNO_3_ in the FGN-182 electrolyte facilitated the reduction of FSI^−^, enabling its involvement in SEI film formation. LiF possesses advantageous properties among solids, characterized by the widest bandgap (13.6 eV) and the broadest electrochemical stability window [49]. Moreover, LiF possesses a remarkably high surface energy (*γ*) with a low diffusion barrier for Li adatoms on its surface [50]. Serving as an effective electron insulator, LiF hampers electron transfer across the interphase and significantly avoids electrolyte consumption and dendrite generation [51].

The N 1*s* spectra of the SEI formed in FGN-182 electrolytes predominantly originate from LiNO_3_, while in the FGN-180, it primarily arises from LiFSI. The incorporation of LiNO_3_ resulted in the enrichment of the SEI with LiN_x_O_y_, contrasting with NSO_2_^−^ in FGN-180 electrolytes (Fig. 4b) [52]. Both LiN_x_O_y_ and Li_3_N, characterized by high ion conductivity (10^−3^ S cm^−1^) [53], enhance the ion diffusion characteristics within the SEI. Following the diffusion–reaction competition principle, rapid Li^+^ diffusion within the SEI film promotes the deposition of spherical Li, well agrees with the analysis of SEI properties. These results also align with the SEM analysis mentioned earlier, indicating that the N-containing SEI coordinates with components like Li_3_N and LiN_x_O_y_, facilitating a more uniform Li^+^ transport. Particularly, the peaks observed at 407.7 and 403.9 eV correspond to NO_3_^−^ and NO_2_^−^, respectively, suggesting incomplete decomposition of LiNO_3_ in the FGN-182 electrolyte at the interface.

Overall, higher levels of N, and F content within the SEI layer are evident in the FGN-182 electrolyte (Figs. 4c and S20c), affirming the collaborative impact of LiFSI and LiNO_3_ on SEI film formation.

### Characterization of Li Morphologies After Cycling

In practical applications, an excess of Li is typically necessary to prevent rapid capacity degradation. Therefore, studying the Li plating/stripping behavior on Li substrates is equally crucial. Consequently, the morphology of Li in Li||Li half cells using FGN-180 and FGN-182 electrolytes after cycling at 0.5 mA cm^−2^ with 1 mAh cm^−2^ was further examined through SEM and atomic force microscopy (AFM).

**Fig. 5:**
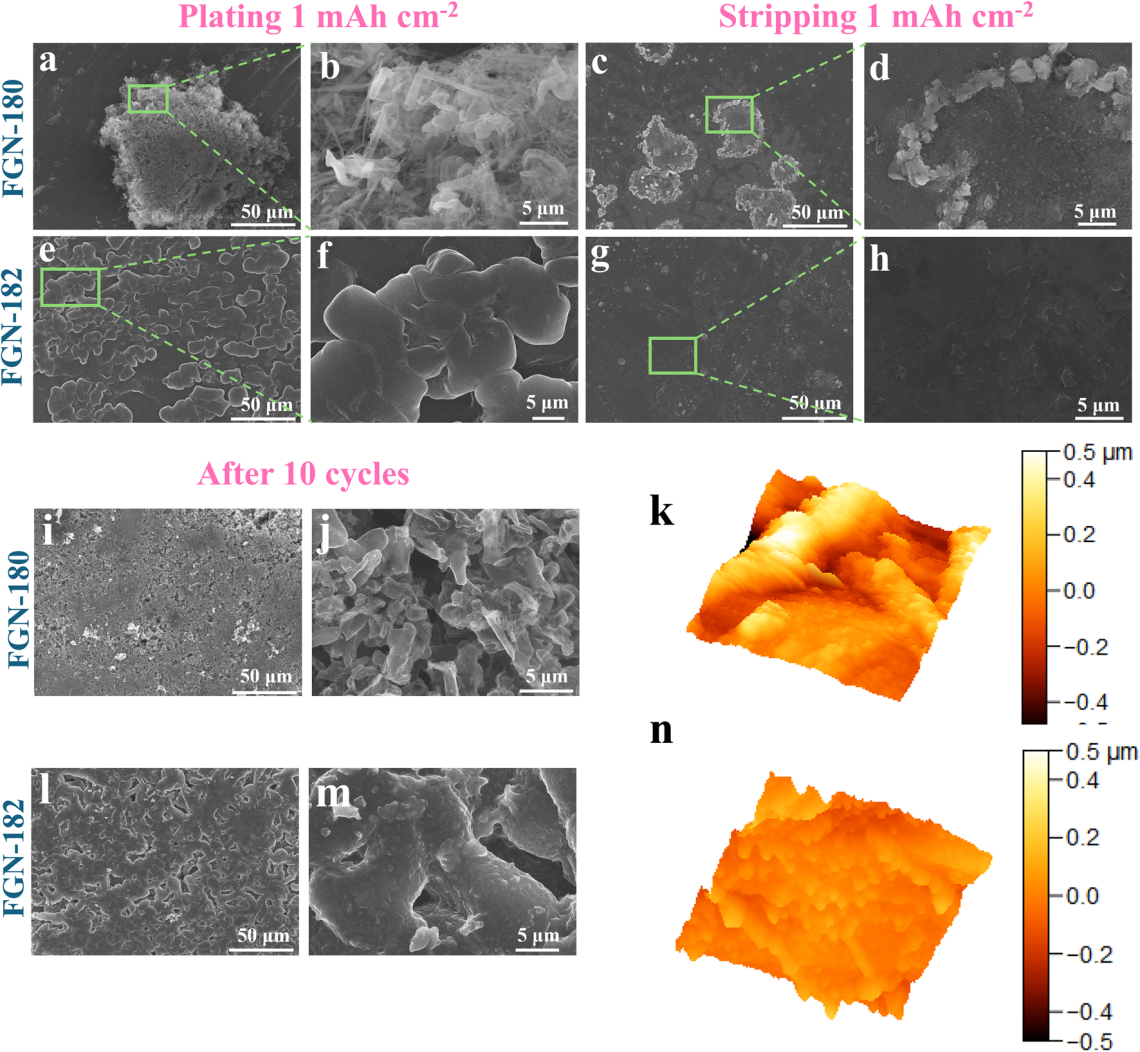
Surface SEM images of symmetric cells using **a**–**d** FGN-180 and **e**–**h** FGN-182 electrolytes after plating and stripping 1 mAh cm^−2^ at a current density of 0.5 mA cm^−2^. The relevant surface SEM and corresponding AFM images after 10 cycles using **i-k** FGN-180 and **l-n** FGN-182 electrolytes

We first investigated the morphology of Li plating/stripping during the initial cycle. It can be observed that in the FGN-180 electrolyte, during the homogenous nucleation process (on the Li substrate, Fig. 5a, b), Li tended to form needle-like structures on the scale of hundreds of nanometers, resulting in a larger specific surface area compared to the micron-level needle-like Li formed during heterogeneous nucleation (on the Cu substrate, Fig. 3a, b). On the Li stripping side, the local stripping of Li in FGN-180 electrolytes resulted in the formation of some pits (Fig. 5c, d). As expected, the synergistic effect of LiFSI and LiNO_3_ in FGN-182 electrolytes effectively regulates the uniform Li deposition due to the formation of a robust SEI film with homogeneous Li^+^ flow, resulting in even larger-scale spherical Li morphologies compared to heterogeneous nucleation (Fig. 5e, f). Moreover, no pits were observed on the stripping side, indicating a uniform stripping process in FGN-182 electrolytes (Fig. 5g, h).

The morphological evolutions of Li were further evaluated after 10 cycles. In FGN-180 electrolytes, Li electrodes exhibited a rougher surface and slightly larger needle-like structures due to the uneven plating/stripping processes (Fig. 5i, j), as well as localized Li protrusions observed in AFM (Fig. 5k). In contrast, FGN-182 electrolytes presented a smooth Li surface (Fig. 5l, m) due to rapid Li^+^ diffusion kinetics and effective regulation of Li deposition, as further confirmed by AFM results (Fig. 5n). After 50 cycles, the Li electrode in FGN-180 electrolytes deteriorated further (Fig. S21a, b), accompanied by dendritic Li growth. In contrast, the Li surface remained densely compact in FGN-182 electrolytes (Fig. S21c, d), devoid of dendritic growth.

These findings indicate that the integration of LiFSI and optimized LiNO_3_ content has facilitated the development of an efficient and highly stable electrolyte (FGN-182), ensuring uniform Li^+^ flow and dendrite-free Li metal anodes.

### Stability Evaluation of NMC111||Li Coin Cells and LCO||Li Pouch Cells

To comprehensively assess the electrochemical performance of the electrolytes, coin cells were constructed utilizing high-loading NCM111 cathodes (2.5 mAh cm^−2^) and Li chips (25 μm, ca. 5 mAh cm^−2^) as anodes. Before full-cell testing, the electrochemical stability of the electrolyte was evaluated using linear sweep voltammetry (LSV). The results indicate that FGN-182 electrolytes achieved a cathodic stability potential as high as 4.6 V (Fig. S22), significantly higher than the FGN-180 which is only around 4 V, suggesting the potential high-voltage batteries employing the FGN-182 electrolyte.

**Fig. 6:**
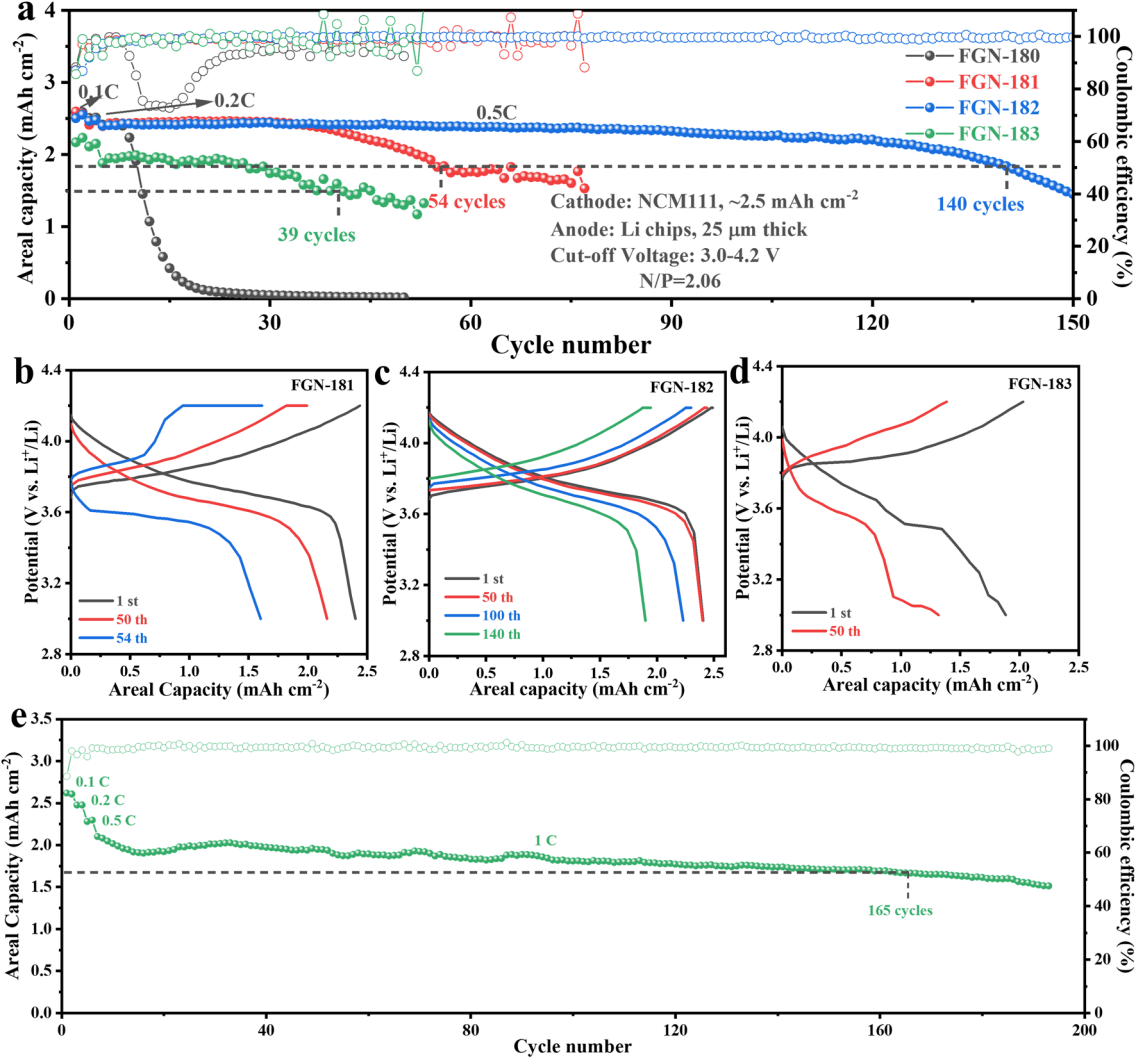
Cycling performance of the NCM111||Li coin cells with different electrolytes at **a** 0.5 C and **e** 1 C at a cutoff voltage range of 3–4.2 V. The charge–discharge curves of NCM111||Li cells in different electrolytes: **b** FGN-181, **c** FGN-182, and **d** FGN-183

In NCM111||Li coin cells employing FGN-182 electrolytes, an impressive cycling stability of 140 cycles was achieved with a capacity retention of 80% at 0.5 C (Fig. 6a). In contrast, cells using FGN-180 electrolytes exhibited a significantly shorter lifespan, lasting only 10 cycles, which is less than a tenth of the performance observed with FGN-182 electrolytes. However, even using FGN-181 and FGN-183 electrolytes, the cycle life of the cells only reached 39 and 54 cycles, respectively. The former is attributed to the relatively low CE, while the latter is due to poorer Li^+^ kinetics caused by higher viscosity.

Figure 6b–d illustrates a comparison of the charge/discharge curves of the different coin cells. As expected, the cells utilizing FGN-182 electrolytes exhibited the lowest electrochemical polarization and only experienced a slight increase in polarization over 140 cycles, indicating superior reversibility in the charge/discharge processes (Fig. 6c). In contrast, the polarization of cells with FGN-181 and FGN-183 electrolytes gradually rises with the cycling process, accompanied by significant capacity degradation (Fig. 6b, d). Furthermore, despite an increased current density (1C), cells employing FGN-182 electrolytes sustained 165 cycles while retaining 80% capacity, as depicted in Fig. 6e.

To evaluate its applicability, high-loading lithium cobalt oxide (LCO) cathodes were further paired with ultrathin Li chips (25 μm) in a 22 × 46 mm^2^ pouch cell configuration, using different electrolytes and various cutoff voltage ranges. The configuration of the pouch cell was illustrated in a prior study [23], where a dual-layer loaded LCO cathode and two Li chips are stacked together to assemble the pouch cell.

**Fig. 7:**
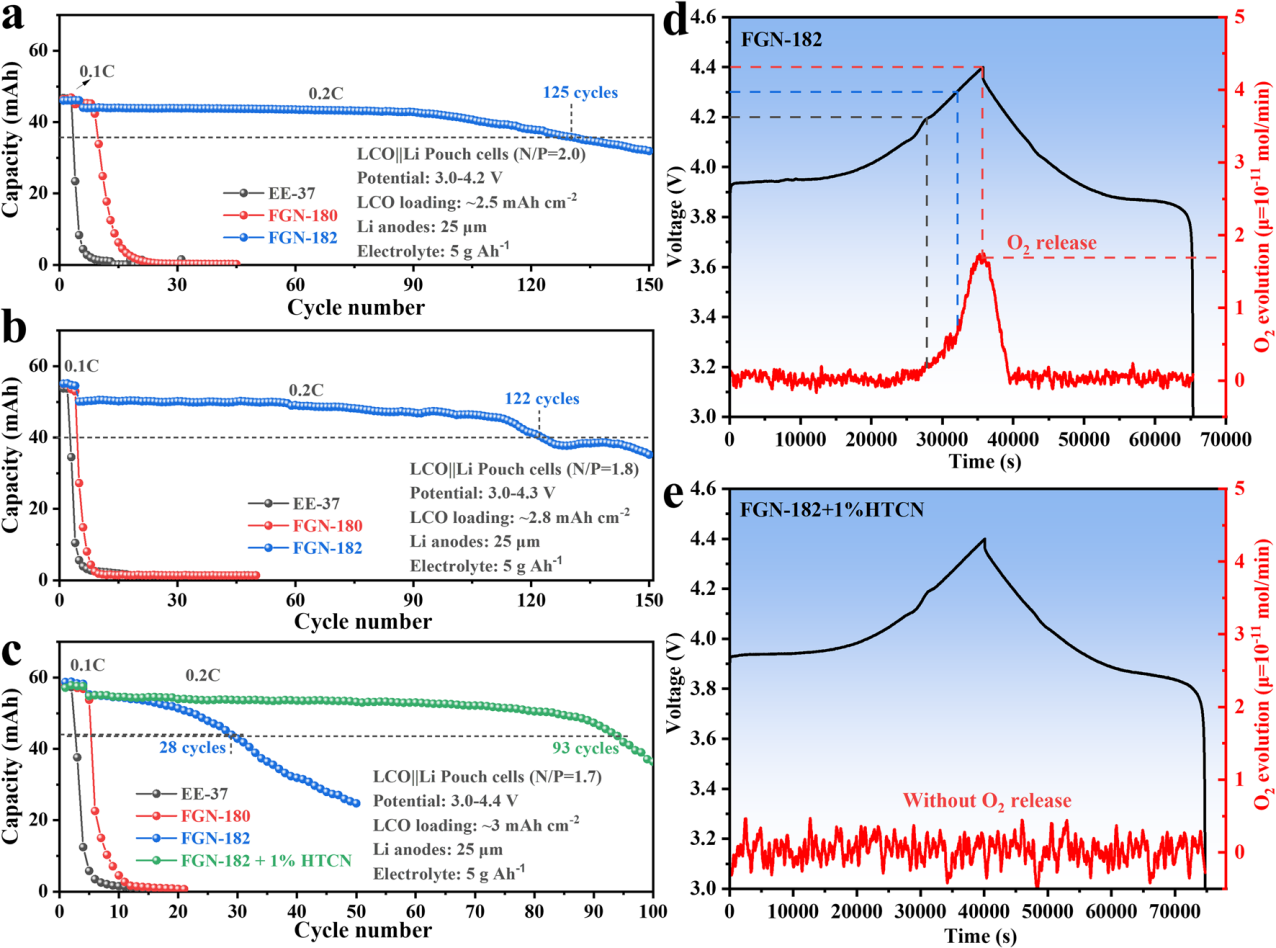
Cycling performance of the LCO||Li pouch cells using different electrolytes at 0.2 C at a cutoff voltage range of **a** 3–4.2 V, **b** 3–4.3 V, and **c** 3–4.4 V. In situ DEMS analysis of the initial charge/discharge cycles for LCO cathodes in **d** FGN-182 and **e** FGN-182 + 1%HTCN electrolytes

Specifically, LCO||Li pouch cells employing FGN-182 electrolytes exhibited remarkable cycling stability, maintaining 80% capacity over 125 cycles at 0.2 C, within a cutoff voltage range of 3–4.2 V (Fig. 7a). Conversely, cells using EE37 and FGN-180 electrolytes exhibited rapid degradation in performance within fewer than 10 cycles due to the unfavorable CE of Li, markedly lower than observed with the FGN-182 electrolyte.

Furthermore, to further explore the cathode stability under high voltage, we extended the voltage to 4.3 and 4.4 V. It is evident that even at 4.3 V, pouch cells using FGN-182 electrolytes can cycle for 122 cycles with 80% capacity retention (Fig. 7b), markedly superior to those with EE37 and FGN-180 electrolytes. However, upon charging to 4.4 V, it was noticeable that cells with FGN-182 electrolytes experienced accelerated capacity decay, maintaining only 80% capacity after 28 cycles. Upon closer examination, we observed gas evolution in pouch cells with FGN-182 electrolytes when charged to 4.4 V, while no such evolution was observed in cells with EE37 and FGN-180 electrolytes under the same conditions (Fig. S23a-c). Moreover, considering the absence of gas evolution in FGN-182 electrolytes when charged to 4.2 V (Fig. S23d), one could speculate that this might be attributed to the decomposition of LiNO_3_ under high voltage, as indicated by the following reaction [54]: ${\text{NO}}_{3}^{ - } \to {\text{NO}}_{{2}} { + }\frac{{1}}{{2}}{\text{O}}_{{2}} {\text{ + e}}^{ - }$.

### FGN-182 with HTCN: Simultaneous Optimization of CEI for High-Voltage Cathode

Addressing the adverse effects of LiNO_3_ in our FGN-182 on the cathode under high voltage, we then introduced the previously reported cathode-improving additive 1,3,6-tricyanohexane (HTCN) [55]. After adding 1% HTCN, the electrolyte (FGN-182 + 1%HTCN) achieved 93 cycles with an 80% capacity retention, notably better than that of FGN-182 electrolytes. This improvement can be ascribed to HTCN stabilizing the cathode electrolyte interphase (CEI) film, thereby preventing the decomposition of LiNO_3_ at high voltage with no gas generation observed (Fig. S23e**)**. To highlight the performance of our full cells (both coin cells and pouch cells), we have included a comparative study of electrolyte modifications in Table S4.

We specifically investigated the influence of HTCN on the Li anode. Figure S24 illustrates the CE of Li using FGN-182 + 1% HTCN electrolytes at a current density of 1 mA cm^−2^ and a capacity of 1 mAh cm^−2^. The results demonstrate that FGN-182 + 1% HTCN electrolytes maintained highly reversible Li plating/stripping, achieving an average CE of 98.6% from the 200th to the 300th cycle. This indicates that the addition of HTCN had a negligible impact on the CE of Li metal.

Furthermore, in situ differential electrochemical mass spectrometry (DEMS) analyses were conducted during the initial charge/discharge processes for the LCO||Li cells in both FGN-182 and FGN-182 + 1%HTCN electrolytes to monitor gas evolution and investigate the failure mechanism of LiNO_3_. As shown in Fig. 7d, the release of O_2_ from the LCO cathode in the FGN-182 electrolyte was observed around 4.2 V during charging. The gas evolution becomes more pronounced above 4.3 V, reaching a peak at 4.4 V, which is consistent with above findings (Fig. S23c). However, NO_2_ was not detected, which slightly deviates from our initial hypothesis. This discrepancy may result from the oxidative decomposition of LiNO_3_ forming more complex nitrogen oxides, necessitating further investigation in the future studies. In contrast, the LCO cathode in the FGN-182 + 1% HTCN electrolyte exhibited a significant suppression of O_2_ evolution. (Fig. 7e). This improvement is attributed to the introduction of HTCN, which forms a stable CEI film, effectively reducing the further oxidative decomposition of LiNO_3_ and enhancing the high-voltage performance of the battery.

DFT calculations were also employed to provide atomic-scale insights. We conducted a comparative analysis of the adsorption energies of LiFSI, LiNO_3_, DEGDME, and HTCN on LiCoO_2_ (104) surfaces, as illustrated in Fig. S25. Specifically, the adsorption energy of HTCN (− 1.02 eV) is lower than those of LiFSI (− 0.21 eV), LiNO_3_ (− 0.88 eV), and DEGDME (− 0.29 eV) on the LiCoO_2_ (104) surface (Fig. S26), suggesting favorable adsorption of HTCN on the LiCoO_2_ surface. This strong adsorption of HTCN effectively hinders bond formation between surface cobalt and other electrolyte molecules, thereby stabilizing the cathode surface.

**Fig. 8:**
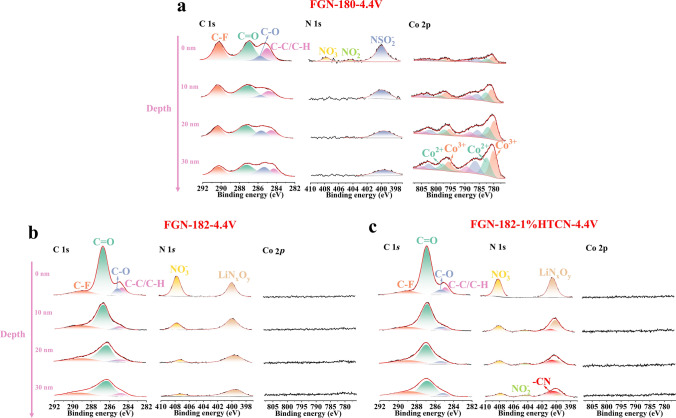
In-depth XPS spectra of the CEI film formed on LCO cycled in **a** FGN-180, **b** FGN-182, and **c** FGN-182–1%HTCN electrolytes within a voltage range of 3–4.4 V

To further explore the failure mechanism of LiNO_3_ under high-voltage conditions and the impact of HTCN on CEI composition, XPS spectra of the cycled LCO electrodes in various electrolytes under different conditions (charged to 4.2 or 4.4 V) were obtained and depicted in Figs. 8 and S27.

Regarding the C 1*s* spectra (Figs. 8 and S27), four primary peaks were observed. The peaks at 290.0 eV (C–F) and 284.8 eV (C–H/C–C) mainly arise from the poly(vinylidene fluoride) binder and conductive carbon, respectively [55]. The peaks at 285.5 eV (C–O) and 286.8 eV (C=O) correspond to by-products resulting from the decomposition of DEGDME solvents. The organic components in all CEIs showed a decrease in intensity with increasing etching depth (from surface to 30 nm). The relatively strong peak of C–F in FGN-180 electrolytes may be attributed to excessive exposure of the cathode without the formation of a well-developed CEI film.

In the N 1*s* spectrum of FGN-180 electrolytes, peaks at 407.6, 404.1, and 399.7 eV correspond to NO_3_^−^, NO_2_^−^, and NSO_2_^−^, originating from FSI^−^ [52]. With etching depths ranging from 10 to 30 nm, only the peak of NSO_2_^–^ was observed in FGN-180 electrolytes. In FGN-182 and FGN-182 + 1%HTCN electrolytes (charged to 4.2 V or 4.4 V), besides residual NO_3_^−^, the formation of LiN_x_O_y_ from LiNO_3_ was also observed, with its content decreasing with etching depth. In the FGN-182 electrolyte (charging to 4.4 V), no NO_2_^−^ was observed even at a depth of 30 nm, which may be attributed to the severe decomposition of LiNO_3_ under high voltage, rendering it incapable of participating in the formation of a stable CEI film. However, no gas evolution phenomenon was observed in FGN-182 electrolytes when charging to only 4.2 V (Fig. S23d), with the signal of NO_2_^−^ can be detected as the etching proceeded due to the partial decomposition of NO_3_^−^ (Fig. S27). Additionally, the –CN peak was only observed in electrolytes containing HTCN (FGN-182 + 1%HTCN), indicating the formation of R–CN–Co in CEI composition due to the strong interaction between HTCN and LiCoO_2_, effectively protecting the cathode and preventing the decomposition of LiNO_3_ under high voltage [55]. This is further supported by the absence of gas generation (Fig. S23e) and the detection of the NO_2_^−^ signal after etching, indicating only partial decomposition of LiNO_3_ (Fig. 8c).

Notably, the Co 2*p* signal was only detectable in FGN-180 electrolytes, displaying two distinct pairs of split peaks at 780.9/796.5 and 782.5/798.1 eV, corresponding to the 2*p*_3/2_ and 2*p*_1/2_ peaks of Co^3+^ and Co^2+^, respectively (Fig. 8a) [56]. This suggests that in the FGN-180 electrolyte, Co^3+^/Co^2+^ dissolution occurs in the cathode due to the undesirable CEI film. An intriguing observation arises when LiNO_3_ was involved in CEI formation, where no Co^3+^/Co^2+^ was detected. This suggests that LiNO_3_ may inhibit the dissolution of Co^3+^/Co^2+^, a phenomenon often overlooked in previous studies. Given the significant benefits of LiNO_3_ to the Li anode, further investigation into its influence on the cathode is meaningful for the development of high-performance LMBs.

In addition, the CEI properties in full cells were further assessed through in situ EIS, as shown in Fig. S28. A three-electrode pouch cell configuration was employed, comprising LCO as the working electrode, Li foil as the counter electrode, and Li wire as the reference electrode. This setup was chosen to mitigate the influence of anode resistance on the cathode. Figure S29 illustrates the variation in CEI resistance (*R*_CEI_) observed in LCO cathodes throughout a complete charge–discharge cycle following initial activation in both FGN-182 and FGN-182 + 1% HTCN electrolytes under different conditions within LCO||Li cells, with these *R*_CEI_ results based on the fitting analysis of the Nyquist plots using the equivalent circuit depicted in Fig. S30. For FGN-182 electrolytes, setting the charge cutoff voltage at 4.2 V resulted in a decrease in *R*_CEI_ during charging due to the formation of the initial CEI on cathodes (Fig. S29, gray symbols**)**. Subsequently, *R*_CEI_ remained stable during the discharging process. Increasing the charge cutoff voltage to 4.3 V caused an overall increase during the charge/discharge process (Fig. S29, red symbols**)**, which may be attributed to slight gas generation. Further increasing the charge cutoff voltage to 4.4 V resulted in a significantly enhanced *R*_CEI_ value in FGN-182 electrolytes, despite a decreasing trend during the charging process (Fig. S29, blue symbols**)**, likely due to the over-decomposition of LiNO_3_ and the gas evolution mentioned above. Conversely, LCO cathodes cycled in FGN-182 + 1%HTCN electrolytes exhibited a significant reduction in *R*_CEI_ compared to FGN-182 throughout the entire charge/discharge process (Fig. S29, green symbols**)**, indicating the formation of a stable CEI attributed to the addition of HTCN. This emphasizes the superiority of FGN-182 + 1%HTCN electrolytes in improving high-voltage LMBs.

## Conclusion

In conclusion, we have developed a promising ether-based electrolyte for high-voltage LMB by incorporating LiNO_3_ (for the anode) and HTCN (for the cathode). A preliminary study has shown that the synergistic effect of LiFSI and LiNO_3_, along with optimized LiNO_3_ content in FGN-182 electrolytes, resulted in a remarkably stable Li metal anode. Specifically, Li||Cu cells delivered over 1400 cycles with CE maintaining around 99% with low polarization. Notably, utilizing Auerbach’s approach, a CE as high as 99.56% can be obtained. Further investigation into the solvation structure revealed strong coordination between NO_3_^−^ and Li^+^, which preferentially participated in the SEI formation on anodes, effectively improving Li deposition. The rapid Li^+^ diffusion kinetics of SEI film in FGN-182 electrolytes was supported by exchange current density and activation energy analysis, facilitating the deposition of spherical Li. Additionally, the N and F-rich SEI using FGN-182 electrolytes was characterized through an in-depth XPS study. The smooth surface of the Li metal anode after cycling in Li||Li symmetrical cells was further collaborated by SEM and AFM results. Subsequently, NCM111||Li full cells were investigated using FGN-182 electrolytes, yielding promising results. In particular, pouch cells featuring high-loading (3 mAh cm^−2^) LCO electrodes, ultrathin Li chips (25 μm) and lean electrolytes (5 g Ah^−1^) exhibited outstanding cycling performance, maintaining 80% capacity after 125 cycles. Moreover, high-voltage LCO||Li (4.4 V) batteries incorporating the cathode additive HTCN (FGN-182 + 1%HTCN) significantly suppress O_2_ evolution resulting from the oxidative decomposition of LiNO_3_, thereby demonstrating stable cycling over 93 cycles. This result provides a workable formula to pursue high Li utilization and high-voltage electrolyte tolerance even for ether-based electrolytes. This study not only optimized the stability of the Li metal anode but also introduced cathode additives to enhance its durability, thus providing valuable insights for the development of high-energy–density LMBs.

## Supplementary Information

Below is the link to the electronic supplementary material.

Supplementary file1 (PDF 3321 KB)

## References

[1] Y. Liang, C. Zhao, H. Yuan, Y. Chen, W. Zhang et al., A review of rechargeable batteries for portable electronic devices. InfoMat **1**, 6–32 (2019). 10.1002/inf2.12000

[2] E.J. Cairns, P. Albertus, Batteries for electric and hybrid-electric vehicles. Annu. Rev. Chem. Biomol. **1**, 299–320 (2010). 10.1146/annurev-chembioeng-073009-100942 10.1146/annurev-chembioeng-073009-100942 22432583

[3] S.S. Rangarajan, S.P. Sunddararaj, A. Sudhakar, C.K. Shiva, U. Subramaniam et al., Lithium-ion batteries—the crux of electric vehicles with opportunities and challenges. Clean Technol. **4**, 908–930 (2022). 10.3390/cleantechnol4040056

[4] Y. Gao, Z. Pan, J. Sun, Z. Liu, J. Wang, High-energy batteries: beyond lithium-ion and their long road to commercialisation. Nano-Micro Lett. **14**, 94 (2022). 10.1007/s40820-022-00844-2 10.1007/s40820-022-00844-2 PMC8986960 35384559

[5] G.E. Blomgren, The development and future of lithium ion batteries. J. Electrochem. Soc. **164**, A5019 (2016). 10.1149/2.0251701jes

[6] N. Nitta, F. Wu, J.T. Lee, G. Yushin, Li-ion battery materials: present and future. Mater. Today **18**, 252–264 (2015). 10.1016/j.mattod.2014.10.040

[7] J. Piątek, S. Afyon, T.M. Budnyak, S. Budnyk, M.H. Sipponen et al., Sustainable Li-ion batteries: chemistry and recycling. Adv. Energy Mater. **11**, 2003456 (2021). 10.1002/aenm.202003456

[8] Z. Luo, X. Qiu, C. Liu, S. Li, C. Wang et al., Interfacial challenges towards stable Li metal anode. Nano Energy **79**, 105507 (2021). 10.1016/j.nanoen.2020.105507

[9] Y. Zhang, T.-T. Zuo, J. Popovic, K. Lim, Y.-X. Yin et al., Towards better Li metal anodes: challenges and strategies. Mater. Today **33**, 56–74 (2020). 10.1016/j.mattod.2019.09.018

[10] D.-H. Liu, Z. Bai, M. Li, A. Yu, D. Luo et al., Developing high safety Li-metal anodes for future high-energy Li-metal batteries: strategies and perspectives. Chem. Soc. Rev. **49**, 54075445 (2020). 10.1039/C9CS00636B 10.1039/c9cs00636b 32658219

[11] R. Wang, W. Cui, F. Chu, F. Wu, Lithium metal anodes: present and future. J. Energy Chem. **48**, 145–159 (2020). 10.1016/j.jechem.2019.12.024

[12] Z. Sun, J. Yang, H. Xu, C. Jiang, Y. Niu et al., Enabling an inorganic-rich interface via cationic surfactant for high-performance lithium metal batteries. Nano-Micro Lett. **16**, 141 (2024). 10.1007/s40820-024-01364-x 10.1007/s40820-024-01364-x PMC10912072 38436814

[13] S. Zhang, G. Yang, Z. Liu, X. Li, X. Wang et al., Competitive solvation enhanced stability of lithium metal anode in dual-salt electrolyte. Nano Lett. **21**, 3310–3317 (2021). 10.1021/acs.nanolett.1c00848 33797262 10.1021/acs.nanolett.1c00848

[14] X. Zheng, L. Huang, W. Luo, H. Wang, Y. Dai et al., Tailoring electrolyte solvation chemistry toward an inorganic-rich solid-electrolyte interphase at a Li metal anode. ACS Energy Lett. **6**, 2054–2063 (2021). 10.1021/acsenergylett.1c00647

[15] W. Chen, C. Zhao, B. Li, Q. Jin, X. Zhang et al., A mixed ether electrolyte for lithium metal anode protection in working lithium-sulfur batteries. Energy Envron. Mater. **3**, 160–165 (2020). 10.1002/eem2.12073

[16] T. Li, X. Zhang, N. Yao, Y. Yao, L. Hou et al., Stable anion-derived solid electrolyte interphase in lithium metal batteries. Angew. Chem. Int. Ed. Engl. **60**, 22683–22687 (2021). 10.1002/anie.202107732 34399018 10.1002/anie.202107732

[17] J. You, S. Zhang, L. Deng, M. Li, X. Zheng et al., Suppressing Li dendrite by a protective biopolymeric film from tamarind seed polysaccharide for high-performance Li metal anode. Electrochim. Acta **299**, 636–644 (2019). 10.1016/j.electacta.2019.01.045

[18] S. Huang, K. Long, Y. Chen, T. Naren, P. Qing et al., In situ formed tribofilms as efficient organic/inorganic hybrid interlayers for stabilizing lithium metal anodes. Nano-Micro Lett. **15**, 235 (2023). 10.1007/s40820-023-01210-6 10.1007/s40820-023-01210-6 PMC10597943 37874415

[19] J. You, H. Deng, X. Zheng, H. Yan, L. Deng et al., Stabilized and almost dendrite-free Li metal anodes by in situ construction of a composite protective layer for Li metal batteries. ACS Appl. Mater. Interfaces **14**, 5298–5307 (2022). 10.1021/acsami.1c20826 35044150 10.1021/acsami.1c20826

[20] C. Chen, Q. Liang, G. Wang, D. Liu, X. Xiong, Grain-boundary-rich artificial SEI layer for high-rate lithium metal anodes. Adv. Funct. Mater. **32**, 2107249 (2022). 10.1002/adfm.202107249

[21] J. You, Y. Hu, X. Han, L. Deng, X. Zheng et al., Highly ion-conducting protective layers with nanomicro engineering for high-performance lithium metal anodes. ACS. Sustain. Chem. Eng. **11**, 13407–13414 (2023). 10.1021/acssuschemeng.3c03017

[22] S. Zhang, J. You, J. Chen, Y. Hu, C. Wang et al., Aluminum-based metal-organic frameworks derived Al_2_O_3_-loading mesoporous carbon as a host matrix for lithium-metal anodes. ACS Appl. Mater. Interfaces **11**, 47939–47947 (2019). 10.1021/acsami.9b16363 31774640 10.1021/acsami.9b16363

[23] S. Zhang, J. You, Z. He, J. Zhong, P. Zhang et al., Scalable lithiophilic/sodiophilic porous buffer layer fabrication enables uniform nucleation and growth for lithium/sodium metal batteries. Adv. Funct. Mater. **32**, 2200967 (2022). 10.1002/adfm.202200967

[24] J. He, A. Manthiram, 3D CoSe@C aerogel as a host for dendrite-free lithium-metal anode and efficient sulfur cathode in Li-S full cells. Adv. Energy Mater. **10**, 2002654 (2020). 10.1002/aenm.202002654

[25] C. Lu, M. Tian, X. Zheng, C. Wei, M. Rummeli et al., Cotton pad derived 3D lithiophilic carbon host for robust Li metal anode: in-situ generated ionic conductive Li_3_N protective decoration. J. Chem. Eng. **430**, 132722 (2022). 10.1016/j.cej.2021.132722

[26] H. Lin, Z. Zhang, Y. Wang, X. Zhang, Z. Tie et al., Template-sacrificed hot fusion construction and nanoseed modification of 3D porous copper nanoscaffold host for stable-cycling lithium metal anodes. Adv. Funct. Mater. **31**, 2102735 (2021). 10.1002/adfm.202102735

[27] Y. Zhang, M. Yao, T. Wang, H. Wu, Y. Zhang, A 3D hierarchical host with gradient-distributed dielectric properties toward dendrite-free lithium metal anode. Angew. Chem. Int. Ed. Engl. **63**, e202403399 (2024). 10.1002/anie.202403399 38483103 10.1002/anie.202403399

[28] Z. Zhang, J. You, S. Zhang, C. Wang, Y. Zhou et al., Metal organic framework nanorod doped solid polymer electrolyte with decreased crystallinity for high-performance all-solid-state lithium batteries. ChemElectroChem **7**, 1125–1134 (2020). 10.1002/celc.201901987

[29] Y. Mu, S. Yu, Y. Chen, Y. Chu, B. Wu et al., Highly efficient aligned ion-conducting network and interface chemistries for depolarized all-solid-state lithium metal batteries. Nano-Micro Lett. **16**, 86 (2024). 10.1007/s40820-023-01301-4 10.1007/s40820-023-01301-4 PMC10786779 38214843

[30] X. Li, Y. Wang, K. Xi, W. Yu, J. Feng et al., Quasi-solid-state ion-conducting arrays composite electrolytes with fast ion transport vertical-aligned interfaces for all-weather practical lithium-metal batteries. Nano-Micro Lett. **14**, 210 (2022). 10.1007/s40820-022-00952-z 10.1007/s40820-022-00952-z PMC9622961 36315314

[31] X. Yang, J. Liu, N. Pei, Z. Chen, R. Li et al., The critical role of fillers in composite polymer electrolytes for lithium battery. Nano-Micro Lett. **15**, 74 (2023). 10.1007/s40820-023-01051-3 10.1007/s40820-023-01051-3 PMC10050671 36976386

[32] K. Wang, Y. Chen, L. Zhang, Q. Zhang, Z. Cheng et al., One step hot-pressing method for hybrid Li metal anode of solid-state lithium metal batteries. J. Mater. Sci. Technol. **153**, 32–40 (2023). 10.1016/j.jmst.2022.12.055

[33] T. Krauskopf, B. Mogwitz, H. Hartmann, D.K. Singh, W.G. Zeier et al., The fast charge transfer kinetics of the lithium metal anode on the garnet-type solid electrolyte Li_6.25_Al_0.25_La_3_Zr_2_O_12_. Adv. Energy Mater. **10**, 2000945 (2020). 10.1002/aenm.202000945

[34] X. Wang, X. Zhang, P. Shi, L. Hou, M. Zhou et al., Glycolide additives enrich organic components in the solid electrolyte interphase enabling stable ultrathin lithium metal anodes. Mater. Chem. Front. **5**, 2791–2797 (2021). 10.1039/D0QM01134G

[35] J. Zhou, B. Hao, M. Peng, L. Zhang, H. Ji et al., Nonafluorobutane-1-sulfonic acid induced local high concentration additive interface for robust SEI formation of high-voltage (5 V-class) lithium metal batteries. Adv. Energy Mater. **13**, 2204174 (2023). 10.1002/aenm.202204174

[36] M. Li, C. Chen, H. Luo, Q. Xu, K. Yan et al., Constructing inorganic-rich solid electrolyte interphase via adjusting electrolyte additives for stable Li metal anodes. J. Mater. Chem. A **12**, 10072–10080 (2024). 10.1039/D3TA07655E

[37] A. Andersen, N.N. Rajput, K.S. Han, H. Pan, N. Govind et al., Structure and dynamics of polysulfide clusters in a nonaqueous solvent mixture of 1, 3-dioxolane and 1, 2-dimethoxyethane. Chem. Mater. **31**, 2308–2319 (2019). 10.1021/acs.chemmater.8b03944

[38] P. Zhang, H. Jin, T. Wang, M. Wang, Insight into the effect of lithium-dendrite suppression by lithium bis(fluorosulfony)imide/1, 2-dimethoxyethane electrolytes. Electrochim. Acta **277**, 116–126 (2018). 10.1016/j.electacta.2018.05.002

[39] G.A. Giffin, The role of concentration in electrolyte solutions for non-aqueous lithium-based batteries. Nat. Commun. **13**, 5250 (2022). 10.1038/s41467-022-32794-z 36068237 10.1038/s41467-022-32794-z PMC9448741

[40] B.D. Adams, J. Zheng, X. Ren, W. Xu, J. Zhang, Accurate determination of coulombic efficiency for lithium metal anodes and lithium metal batteries. Adv. Energy Mater. **8**, 1702097 (2018). 10.1002/aenm.201702097

[41] T. Chen, J. You, R. Li, H. Li, Y. Wang et al., Ultra-low concentration electrolyte enabling LiF-rich SEI and dense plating/stripping processes for lithium metal batteries. Adv. Sci. **9**, 2203216 (2022). 10.1002/advs.202203216 10.1002/advs.202203216 PMC9534938 35978270

[42] J. Fu, X. Ji, J. Chen, L. Chen, X. Fan et al., Lithium nitrate regulated sulfone electrolytes for lithium metal batteries. Angew. Chem. Int. Ed. Engl. **132**, 22378–22385 (2020). 10.1002/anie.202009575 10.1002/anie.202009575 32841474

[43] X. Chen, Y. Yao, C. Yan, R. Zhang, X. Cheng et al., A diffusion-reaction competition mechanism to tailor lithium deposition for lithium-metal batteries. Angew. Chem. Int. Ed. Engl. **59**, 7743–7747 (2020). 10.1002/anie.202000375 32160379 10.1002/anie.202000375

[44] Z. Wang, F. Qi, L. Yin, Y. Shi, C. Sun et al., An anion-tuned solid electrolyte interphase with fast ion transfer kinetics for stable lithium anodes. Adv. Energy Mater. **10**, 1903843 (2020). 10.1002/aenm.201903843

[45] K.-E. Kim, J.Y. Jang, I. Park, M.-H. Woo, M.-H. Jeong et al., A combination of lithium difluorophosphate and vinylene carbonate as reducible additives to improve cycling performance of graphite electrodes at high rates. Electrochem. Commun. **61**, 121–124 (2015). 10.1016/j.elecom.2015.10.013

[46] S.-J. Zhang, Z.-Y. Yin, Z.-Y. Wu, D. Luo, Y.-Y. Hu et al., Achievement of high-cyclability and high-voltage Li-metal batteries by heterogeneous SEI film with internal ionic conductivity/external electronic insulativity hybrid structure. Energy Storage Mater. **40**, 337–346 (2021). 10.1016/j.ensm.2021.05.029

[47] C. Yan, Y.-X. Yao, X. Chen, X.-B. Cheng, X.-Q. Zhang et al., Lithium nitrate solvation chemistry in carbonate electrolyte sustains high-voltage lithium metal batteries. Angew. Chem. Int. Ed. **57**, 14055–14059 (2018). 10.1002/anie.201807034 10.1002/anie.201807034 30094909

[48] J. Zheng, P. Yan, D. Mei, M.H. Engelhard, S.S. Cartmell et al., Highly stable operation of lithium metal batteries enabled by the formation of a transient high-concentration electrolyte layer. Adv. Energy Mater. **6**, 1502151 (2016). 10.1002/aenm.201502151

[49] W.D. Richards, L.J. Miara, Y. Wang, J.C. Kim, G. Ceder, Interface stability in solid-state batteries. Chem. Mater. **28**, 266–273 (2016). 10.1021/acs.chemmater.5b04082

[50] Y. Ozhabes, D. Gunceler, T. Arias, Stability and surface diffusion at lithium-electrolyte interphases with connections to dendrite suppression (2015), Preprint at arXiv:1504.05799. 10.48550/arXiv.1504.05799

[51] F. Li, J. He, J. Liu, M. Wu, Y. Hou et al., Gradient solid electrolyte interphase and lithium-ion solvation regulated by bisfluoroacetamide for stable lithium metal batteries. Angew. Chem. Int. Ed. **60**, 6600–6608 (2021). 10.1002/anie.202013993 10.1002/anie.202013993 33306226

[52] Y. Liang, W. Wu, D. Li, H. Wu, C. Gao et al., Highly stable lithium metal batteries by regulating the lithium nitrate chemistry with a modified eutectic electrolyte. Adv. Energy Mater. **12**, 2202493 (2022). 10.1002/aenm.202202493

[53] H. Xu, Y. Li, A. Zhou, N. Wu, S. Xin et al., Li_3_N-modified garnet electrolyte for all-solid-state lithium metal batteries operated at 40 ℃. Nano Lett. **18**, 7414–7418 (2018). 10.1021/acs.nanolett.8b03902 30352159 10.1021/acs.nanolett.8b03902

[54] T. Broder, D. Silvester, L. Aldous, C. Hardacre, A. Crossley et al., The electrochemical oxidation and reduction of nitrate ions in the room temperature ionic liquid [C_2_mim][NTf_2_]; the latter behaves as a ‘melt’ rather than an ‘organic solvent.’ New J. Chem. **31**, 966–972 (2007). 10.1039/B701097D

[55] X. Yang, M. Lin, G. Zheng, J. Wu, X. Wang et al., Enabling stable high-voltage LiCoO_2_ operation by using synergetic interfacial modification strategy. Adv. Funct. Mater. **30**, 2004664 (2020). 10.1002/adfm.202004664

[56] L. Feng, Z. Yin, C. Wang, Z. Li, S. Zhang et al., Glassy/ceramic Li_2_TiO_3_/LixByOz analogous “solid electrolyte interphase” to boost 4.5 V LiCoO_2_ in sulfide-based all-solid-state batteries. Adv. Funct. Mater. **33**, 2210744 (2023). 10.1002/adfm.202210744

