# Supplementary material for: Boosting High-Voltage Practical Lithium Metal Batteries with Tailored Additives
Source: Nanomicro Lett. 2024 Jul 29;16:257. doi: 10.1007/s40820-024-01479-1 (PMC11286617; doi:10.1007/s40820-024-01479-1)
Supplement: Supplementary file 1 — Supplementary file1 (PDF 3321 KB) [file 40820_2024_1479_MOESM1_ESM.pdf]

Supporting Information for

Boosting High-Voltage Practical Lithium Metal Batteries with Tailored Additives

Jinhai You[1,3,#], Qiong Wang[4,6,#], Runhong Wei[3,#], Li Deng[4], Yiyang Hu[1], Li Niu[3], Jingkai Wang[2], Xiaomei Zheng[2,*], Junwei Li[5,*], Yao Zhou[1,*], Jun-Tao Li[1,*]

[1] College of Energy, Xiamen University, Xiamen University, Xiamen 361005, P. R. China

[2] Magnetism Key Laboratory of Zhejiang Province, College of Materials and Chemistry, China Jiliang University, Hangzhou 310018, P. R. China

[3] Laboratory for Soft Matter and Biophysics, Department of Physics and Astronomy, KU Leuven, Leuven 3001, Belgium

[4] State Key Lab of Physical Chemistry of Solid Surface, College of Chemistry and Chemical Engineering, Xiamen University, Xiamen 361005, P. R. China

[5] Department of Chemical Engineering, KU Leuven, Celestijnenlaan 200F, B-3001 Leuven, Belgium

[6] College of Chemistry and Chemical Engineering, Northwest Normal University, Lanzhou, Gansu 730070, P. R. China

[#] Jinhai You, Qiong Wang, and Runhong Wei contributed equally to this paper.

*Corresponding authors. E-mail: zheng_xiaomei@cjlu.edu.cn (Xiaomei Zheng); junwei.li@kuleuven.be (Junwei Li); zhouy@xmu.edu.cn (Yao Zhou); jtli@xmu.edu.cn (Jun-Tao Li)

S1 Experimental Sections

S1.1 Materials and chemicals

Lithium Bis(fluorosulfonyl)imide (LiFSI) was supported by Guangdong Canrd New Energy Technology Co.,Ltd., China. $LiNO_3$ and 1,3,6-Tricyanohexane (HTCN) were purchased from Shanghai Aladdin Reagent Co., Ltd., China. Diethylene glycol dimethyl ether (DEGDME) was provided by alfa aesar chemical co. Ltd., China. The battery-grade electrolyte 1 M LiTFSI in 1,2-dimethoxyethane (DME)/1,3-dioxolane (DOL) (DME:DOL=1/1 v/v) and 1 M $LiPF_6$ in ethylene carbonate (EC)/ethyl methyl carbonate (EMC) (EC:EMC=3/7 v/v) was supplied by Suzhou Fosai Regent Co., Ltd. The Li chips (>99.9%, thickness: 25 μm, substrate: 15 μm Cu foils) was brought from China Energy Lithium Co., Ltd. The solvent was dewatered by molecular sieve for a week before use.

S1.2 Preparation of electrolytes

Mixture electrolytes of LiFSI, DEGDME and $LiNO_3$ with molar ratios of 1:8:x, where x is 0, 0.2, 0.4, 0.8, 1, 2, and 3 respectively. As a comparison, DEGDME and $LiNO_3$ with ratios of 8:1, 8:3, 8:5 and saturated $LiNO_3$ in DEGDME also prepared. The FGN-182+1%HTCN electrolyte is formulated by incorporating 1% of HTCN into FGN-182 electrolytes.

S1.3 Preparation of $LiNi_{1/3}Co_{1/3}Mn_{1/3}O_2$ (NCM111) and $LiCoO_2$ (LCO) cathodes

For the coin cells, the NCM111 powder, carbon black, PVDF with a weight ratio of 90:4:6 was mixed and stirred for 12 h to form homogeneous slurry. The slurry was further casted on Al foils and following dried in vacuum at 80 °C for 12 h and roll-in treatment to acquire NCM111 cathode electrodes. For the pouch cells, the dual-layer coated LCO cathodes was provided by Contemporary Amperex Technology Limited.

S1.4 Electrochemical tests

The electrochemical performance of the electrodes was tested in coin cells (CR2025) which were assembled in an Ar-filled glove box (O_2 < 0.01 ppm, H_2O < 0.01 ppm). The separator here was polypropylene film (Celgard 2400). The conventional Coulombic efficiency (CE) of Li was measured in Li||Cu cells. Before the test, the Li||Cu cells were first discharged at a current density of 0.5 mA cm^{-2} for 8 h and charged to 1.2 V to strip all of the plating Li to stabilize the initial SEI film. During the tests, a controllable amount of Li was plated on the Cu foils at a constant current density, and a fixed voltage was carried out to strip the Li from the Cu foils. The CE of Li is defined as the ratio of the amount of Li during the stripping process and plating process. The innovative CE of Li was also measured in Li||Cu cells according to Auerbach's test. The Li||Cu cells were activated by the as-mentioned method to stabilize the initial SEI film, and plating a certain amount of Li (depending on the excess amount of Li) on Cu foils to form Li||Cu@Li cells, then charging/discharging at 0.5 mA cm^{-2} for 2 h for 10 cycles, stripping all of the plating Li to calculate the CE, which is defined as:

$$CE=1-\frac{A-B}{10}/C \tag{S1}$$

Where A is the first plating amount of Li on Cu foils, B is the last stripping amount of Li from Cu foils, and C is the amount of charging/discharging process.

The NCM111||Li coin cells were first activated at each lower current density for 2 cycles with a voltage range from 3.0 to 4.2 V and then tested the long-term cycling performance at higher current density. The ratio of negative/positive (N/P) of the cells was 2.06. Similarly, the LCO||Li pouch cells were also activated before long-term cycling. The ratio of N/P is decreased from 2.0 to 1.7 due to the charging cutoff voltage (increase from 4.2 to 4.4 V). The voltage windows for LCO||Li pouch cells were from 3.0 to the different cutoff voltage mentioned. In addition, the amount of electrolyte is fixed at 5 g Ah^{-1}.

The electrochemical impedance spectra (EIS) and linear sweep voltammetry (LSV) experiments of the cells were tested on a CHI660E (Chenghua) electrochemical

analytical instrument. The EIS operating frequency ranged from 10^{-2} to 10^5 Hz, and the LSV profiles were obtained in the voltage range of open-circuit to 5.5 V at a scan rate of 5 mV s^{-1}. The Li^+ transference number (t_{Li+}) was determined using chronoamperometry (applying a DC voltage of 10 mV) and EIS (with frequencies ranging from 10^{-2} to 10^5 Hz) in Li||Li symmetric cells with various electrolytes. The t_{Li+} was obtained according to the Bruce-Vincent equation [S1]:

$$t_{Li^+}=\frac{I_{SS}(\Delta V\text{-}I_0R_0)}{I_0(\Delta V\text{-}I_{SS}R_0)} \tag{S2}$$

where ΔV denotes the voltage polarization applied. I_0 and I_{ss} are the initial and steady-state currents of the cell. R_0 and R_{ss} are the initial and steady-state resistances, respectively.

S1.5 Characterizations

A field emission scanning electron microscope (FESEM, Hitachi S-4800) was used to observe the morphologies of plating Li on Cu foil and Li anodes before and after cycles. The sample was transferred from the argon-filled atmosphere to the SEM vacuum chamber in an instant. Transmission electron microscopy (TEM, JEOL JEM-2100) was conducted to analyze the detailed structural information of materials. X-ray photoelectron spectroscopy (XPS, PHI 5000 VB III) was carried out to investigate the surface information of the materials, and the binding energies reported herein were corrected with reference to the C-C/C-H signal at 284.8 eV. An Al Kα monochromatized radiation (hν=1486.6 eV) was employed as an X-ray source. Atomic force microscopy (AFM) tests were obtained by SPM 5500 (Put in an Ar-filled glove box). The gas evolution of the samples was monitored using in situ differential electrochemical mass spectrometry (DEMS) (Linglu Instruments, Shanghai, i-DEMS QMG220).

S1.6 Calculation methods

The density functional theory (DFT) calculations were conducted utilizing the PW and PP modules incorporated in the Quantum ESPRESSO distribution [S2]. Interactions among nuclei and core electrons were described employing the ultrasoft method within the framework of the generalized gradient approximation (GGA) using the Perdew-Burke-Ernzerhof (PBE) functional. A kinetic energy cutoff of 30 Ry with a charge-density cutoff of 300 Ry was employed to ensure energy convergence. Fermi-surface effects were addressed using the Methfessel-Paxton smearing technique with a smearing parameter of 0.02 Ry. Prior to calculation, models of DME, DEGDME, LiFSI, and $LiNO_3$ were relaxed to optimize their structures. A $LiCoO_2$ slab model with four layers was chose to simulate the cathode $LiCoO_2$. Both lower layers were fixed to simulate the phase of $LiCoO_2$, others were relaxed to simulate the surface. The vacuum layer of 15 Å was added in z axis to avoid the influence from periodic slabs. K-point sampling in the Brillouin zones was conducted using the gamma point. Van der Waals corrections were incorporated using the DFT-D3 method. The molecular dynamics simulations were performed using the GROMACS 2023.02 software package [S3], with all molecules described using the Oplsaa force field [S4]. The initial configurations

of all simulation systems were obtained by uniformly mixing the components of the system using the Packmol software package [S5]. Firstly, energy minimization was conducted for 3000 steps using the steepest descent method to eliminate unreasonable atomic overlaps. Subsequently, relaxation was performed for 100 ps at 298.15 K under both NVT and NPT ensembles, with an integration time step of 1.0 fs. Finally, production simulations were conducted for 50 ns under the NPT ensemble. In the MD simulations, periodic boundary conditions were applied in all directions, with a time step of 2.0 fs. The temperature was maintained at 298.15 K using the V-rescale thermostat with a coupling time of 0.5 ps, and the pressure was controlled at 1 bar using the Parrinello-Rahman barostat with a coupling time of 2.0 ps. All hydrogen-containing bonds (C–H, O–H) were constrained using the LINCS algorithm. The long-range electrostatic interactions were treated with the particle mesh Ewald (PME) method, with a cutoff of 1.2 nm applied to calculate short-range electrostatic and van der Waals interactions. Trajectory analysis was performed using the GROMACS software package.

S2 Supplementary Figures and Tables

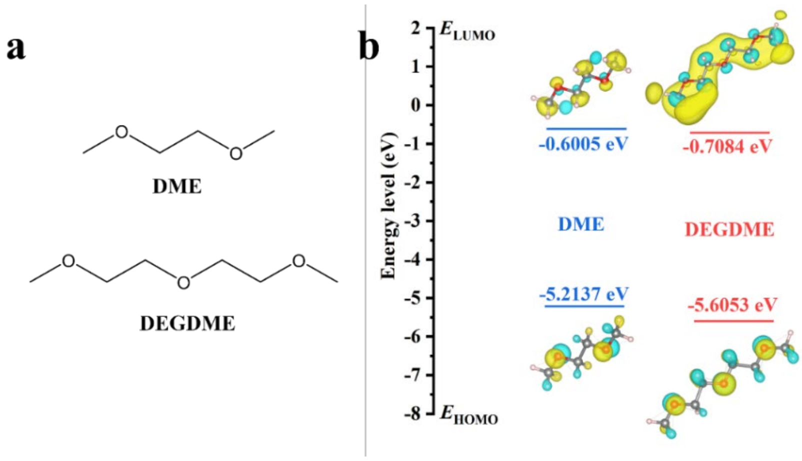

Fig. S1 (**a**) Chemical structure of DEM and DEGDME. (**b**) Diagram of the HOMO and LUMO energy levels of DME and DEGDME

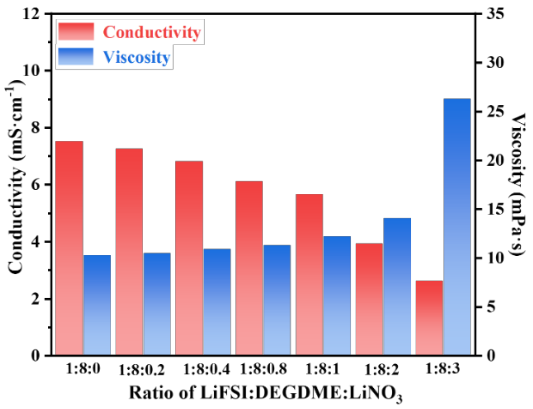

Fig. S2 Conductivity and viscosity of different electrolyte with varying the ration of LiFSI:DEGDME:$LiNO_3$

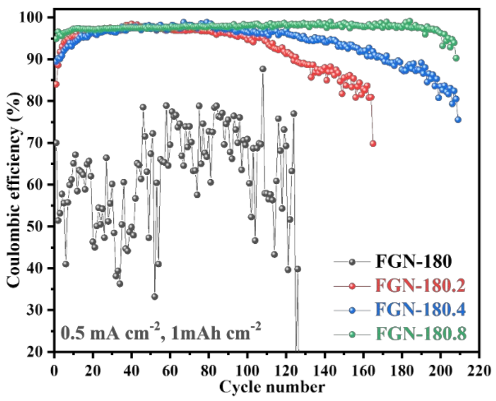

Fig. S3 CE of Li||Cu cells for FGN-180, FGN-180.2, FGN-180.4 and FGN-180.8 electrolytes

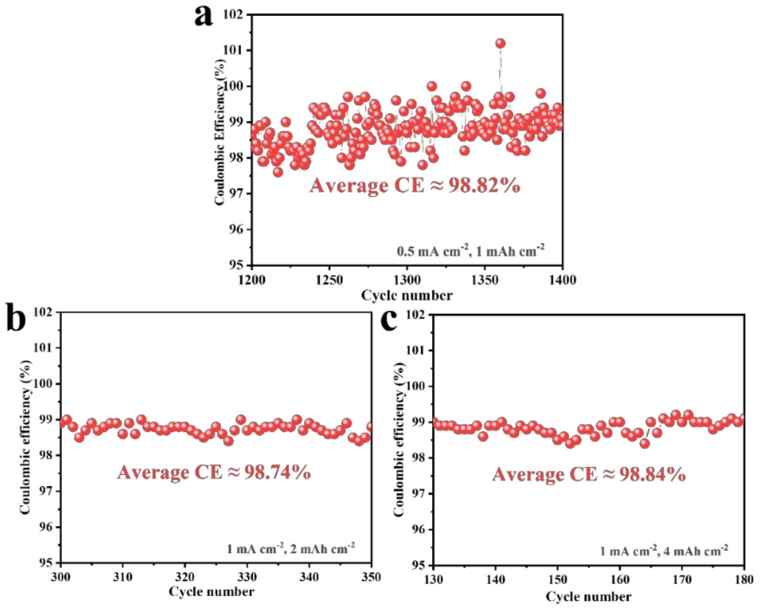

Fig. S4 The average CE of FGN-182 electrolytes under different test conditions in different cycle number intervals.

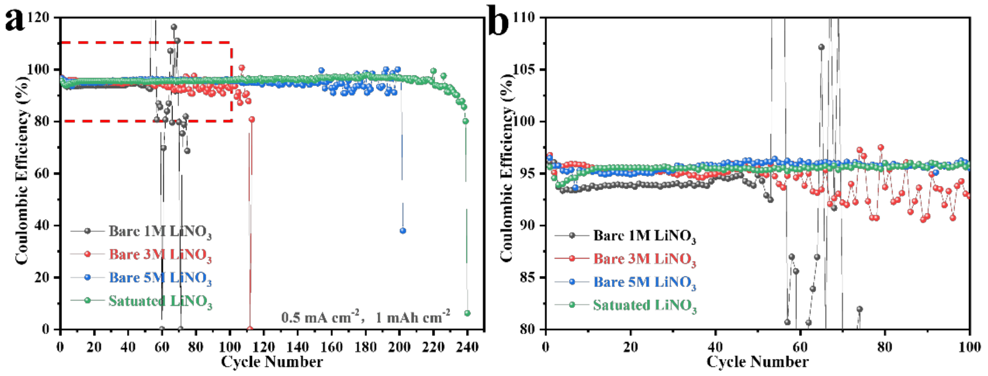

Fig. S5 (**a**) CE of Li||Cu cells for different concentrations of LiNO3 in DEGDME. (**b**) Partial enlarged drawing of (**a**)

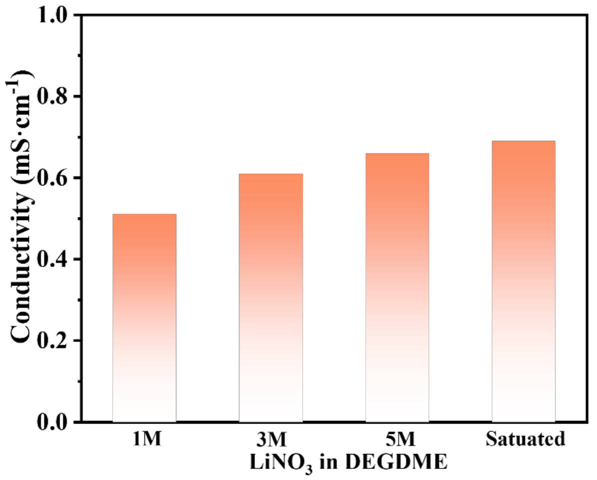

Fig. S6 Conductivity of different concentration $LiNO_3$ in DEGDME

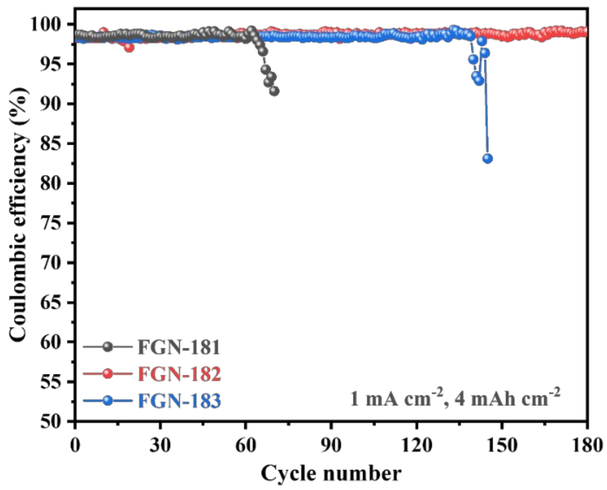

Fig. S7 CE of Li||Cu cells for FGN-181, FGN-182, and FGN-183 electrolytes at 1 mA cm^{-2}, 4 mAh cm^{-2}

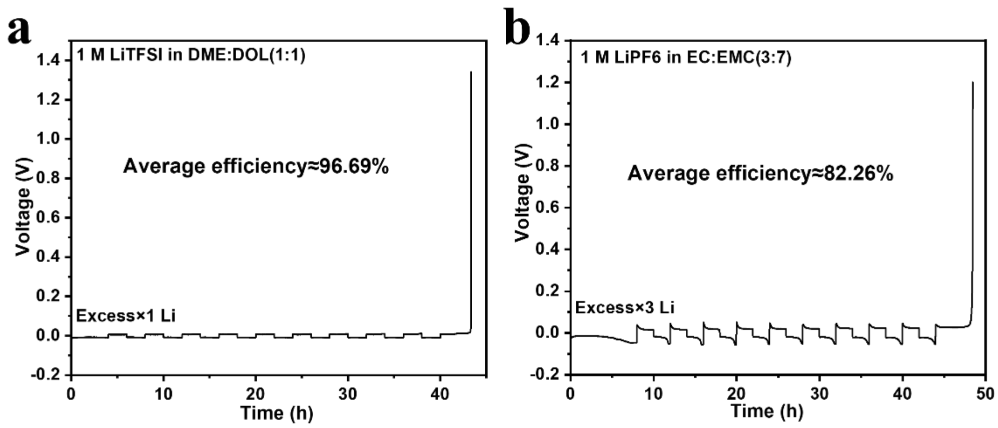

Fig. S8 CE of Li plating/stripping in (**a**) conventional ether-based and (**b**) carbonate-based electrolytes using the Aurbach's method

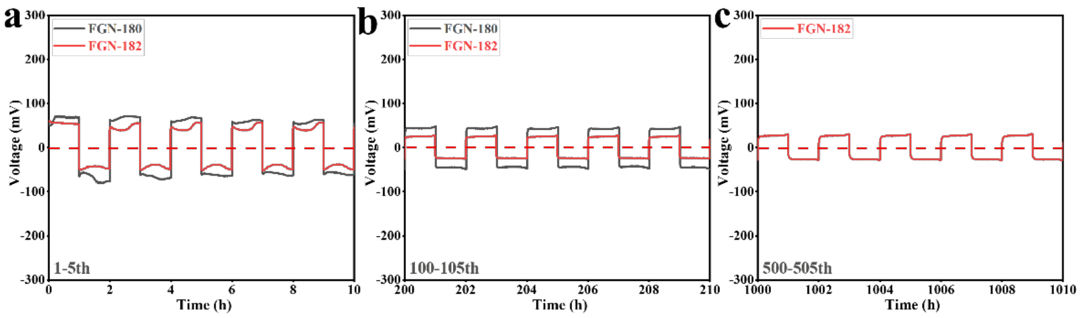

Fig. S9 Detailed analysis of Li||Li cells voltage profiles at a current density of 1.0 mA cm^{-2} with a capacity of 1.0 mAh cm^{-2} in FGN-180 and FGN-182 electrolytes

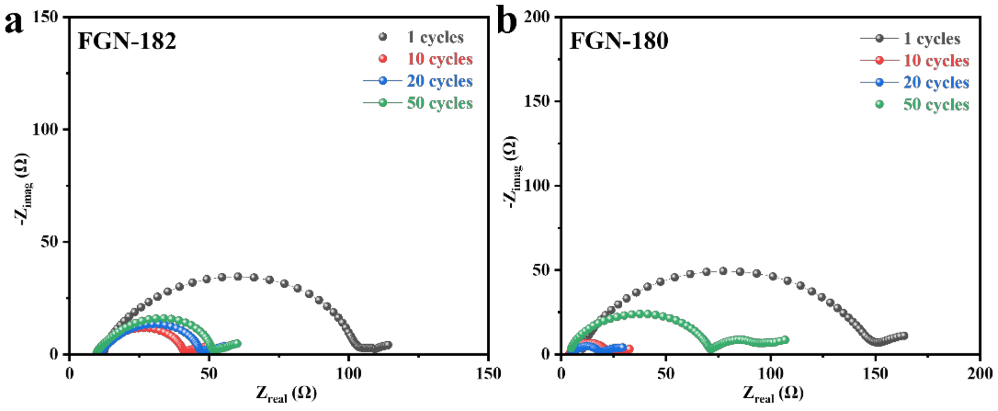

Fig. S10 The Nyquist plots of Li||Li symmetric cells in (**a**) FGN-180 and (**b**) FGN-182 electrolytes after different cycles at 1 mA cm^{-2}, 1 mAh cm^{-2}

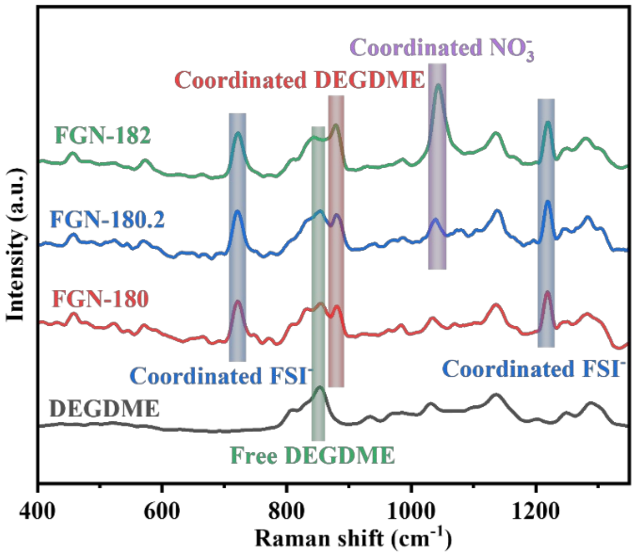

Fig. S11 Raman spectra of DEGDME, FGN-180, FGN-180.2 and FGN-182 electrolytes.

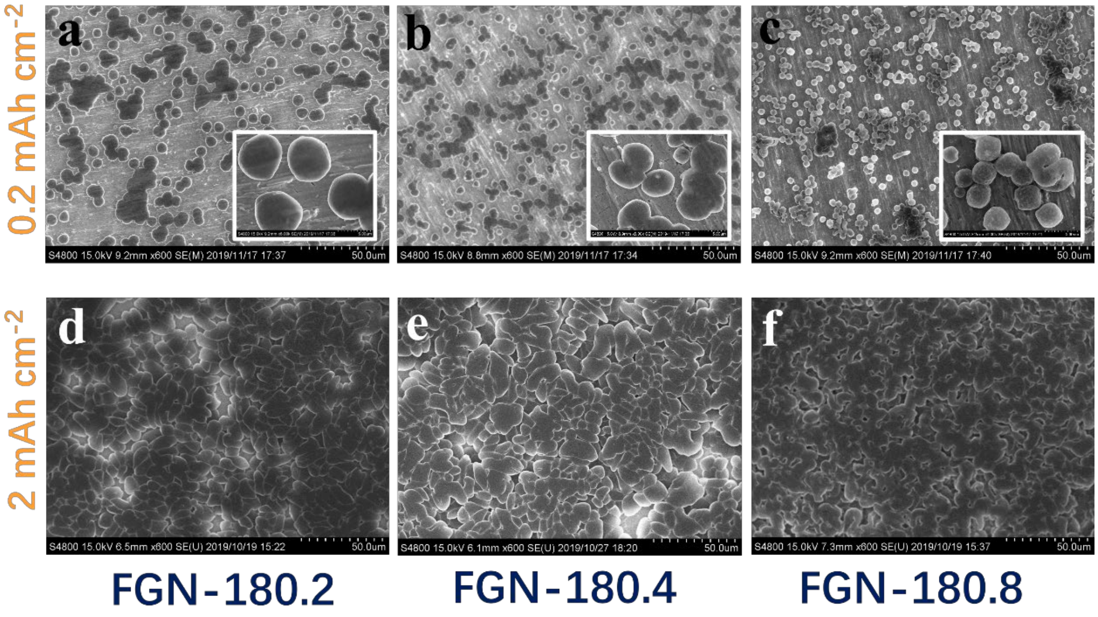

Fig. S12 SEM images of Li metal deposited onto a Cu substrate on top view for (**a, d**) FGN-180.2, (**b, e**) FGN-180.4 and (**c, f**) FGN-180.8 electrolytes with capacities of 0.2 mAh cm^{-2} and 2 mAh cm^{-2}

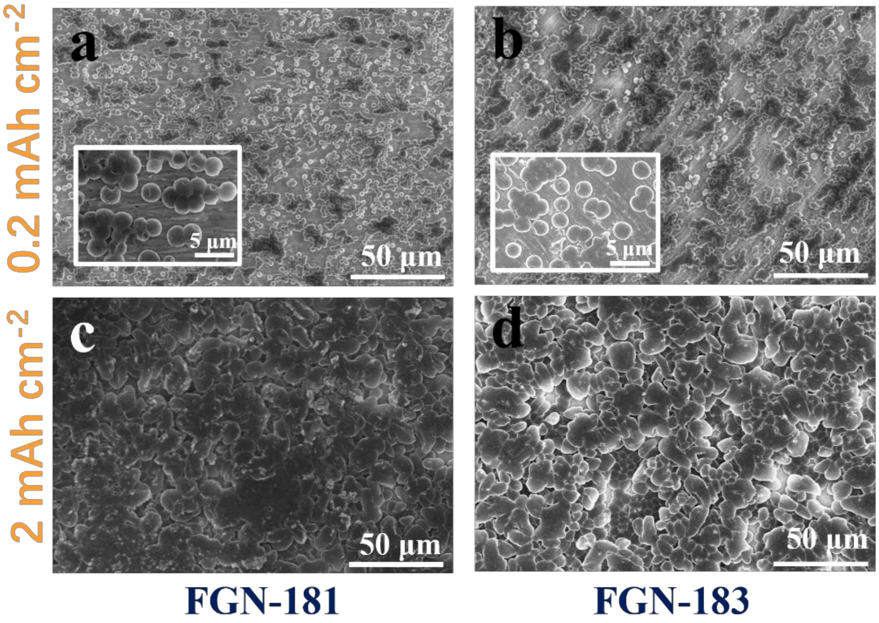

Fig. S13 SEM images of Li metal deposited onto a Cu substrate on top view for (**a, c**) FGN-181 and (**b, d**) FGN-183 electrolytes with capacities of 0.2 mAh cm^{-2} and 2 mAh cm^{-2}

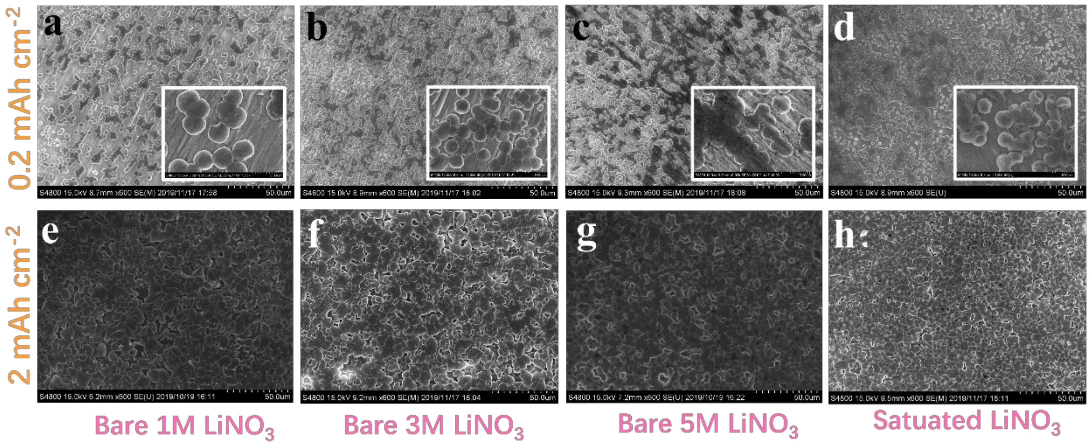

Fig. S14 SEM images of Li metal deposited onto a Cu substrate on top view for (**a, e**) 1 M $LiNO_3$, (**b, f**) 3 M $LiNO_3$ and (**c, g**) 5 M $LiNO_3$ and (**d, h**) saturated $LiNO_3$ electrolytes with capacities of 0.2 mAh cm^{-2} and 2 mAh cm^{-2}

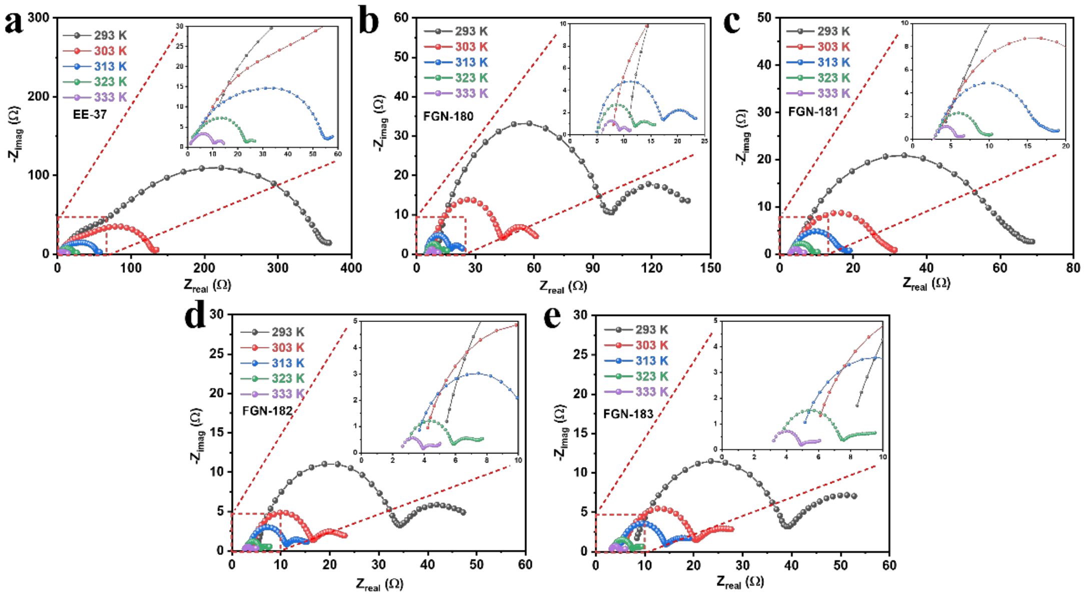

Fig. S15 The Nyquist plots at a temperature range from 20 to 60 °C of Li||Li symmetric cells in (**a**) EE-37, (**b**) FGN-180, (**c**) FGN-181, (**d**) FGN-182 and (**e**) FGN-183 electrolytes after 5 cycles at 1 mA cm^{-2}, 1 mAh cm^{-2}

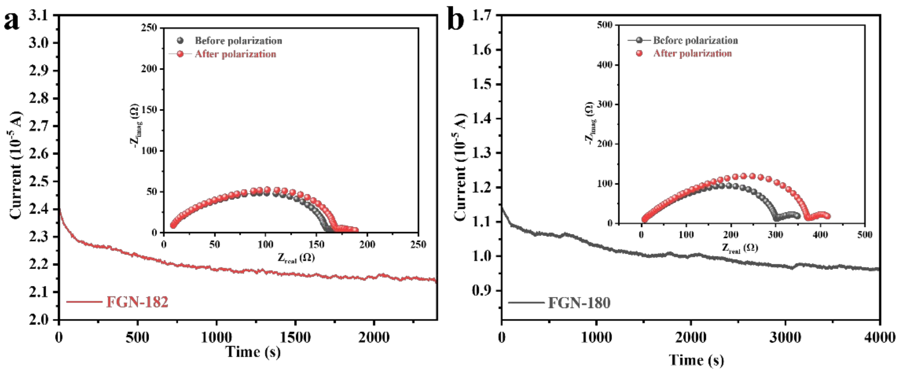

Fig. S16 Current-time profile of symmetrical cells using (**a**) FGN-182 and (**b**) FGN-180 electrolytes. Insets in (**a**) and (**b**) are the impedance spectra before and after potential polarization

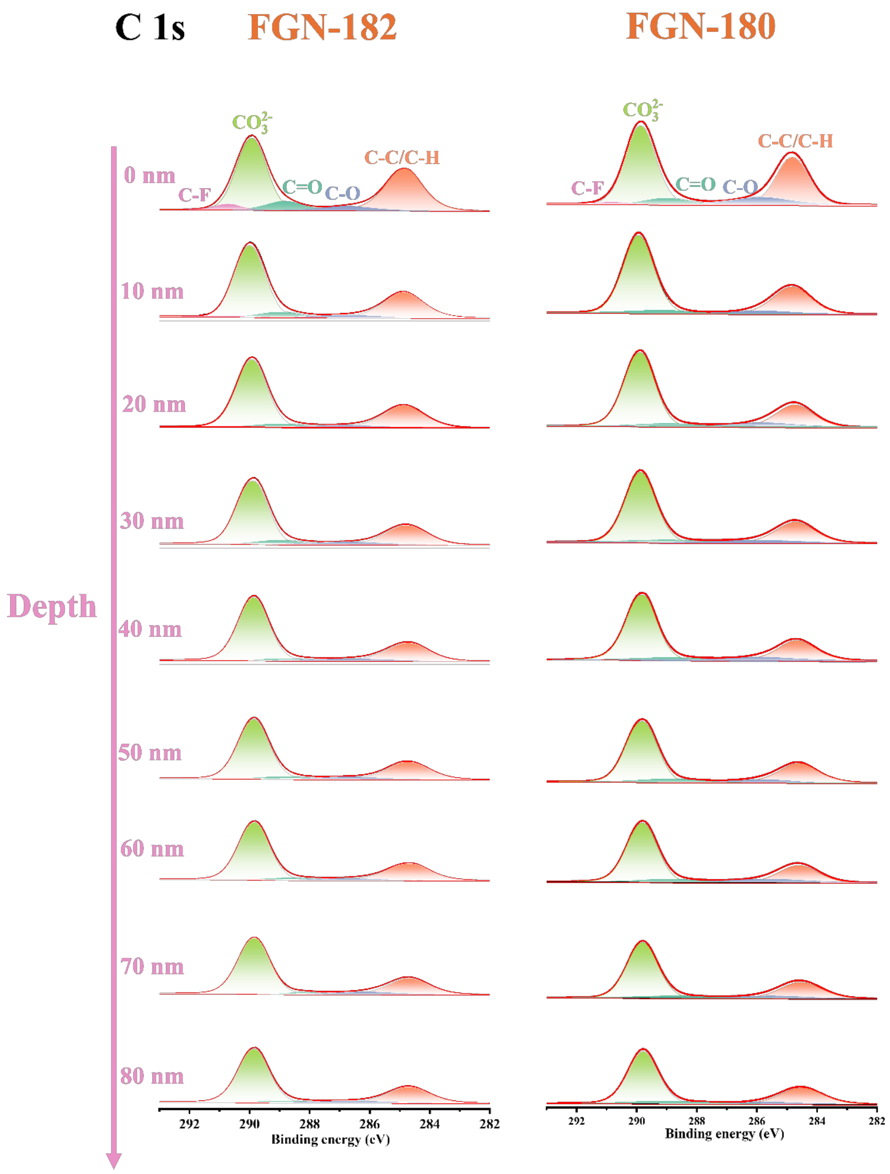

Fig. S17 The in-depth XPS spectra of SEI layer formed on Li metal cycled in FGN-182 and FGN-180 electrolytes of C 1s spectra

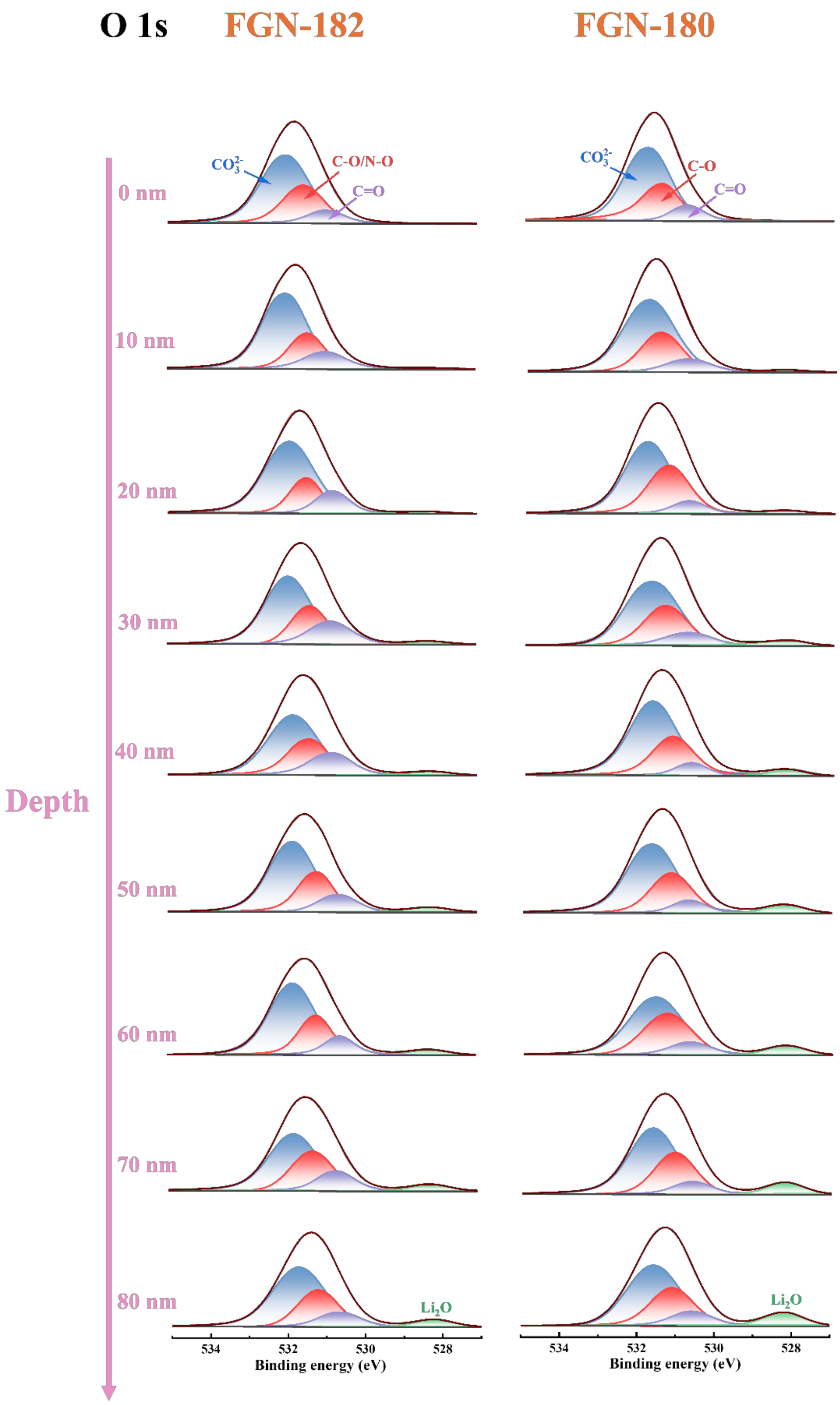

Fig. S18 The in-depth XPS spectra of SEI layer formed on Li metal cycled in FGN-182 and FGN-180 electrolytes of O 1s spectra

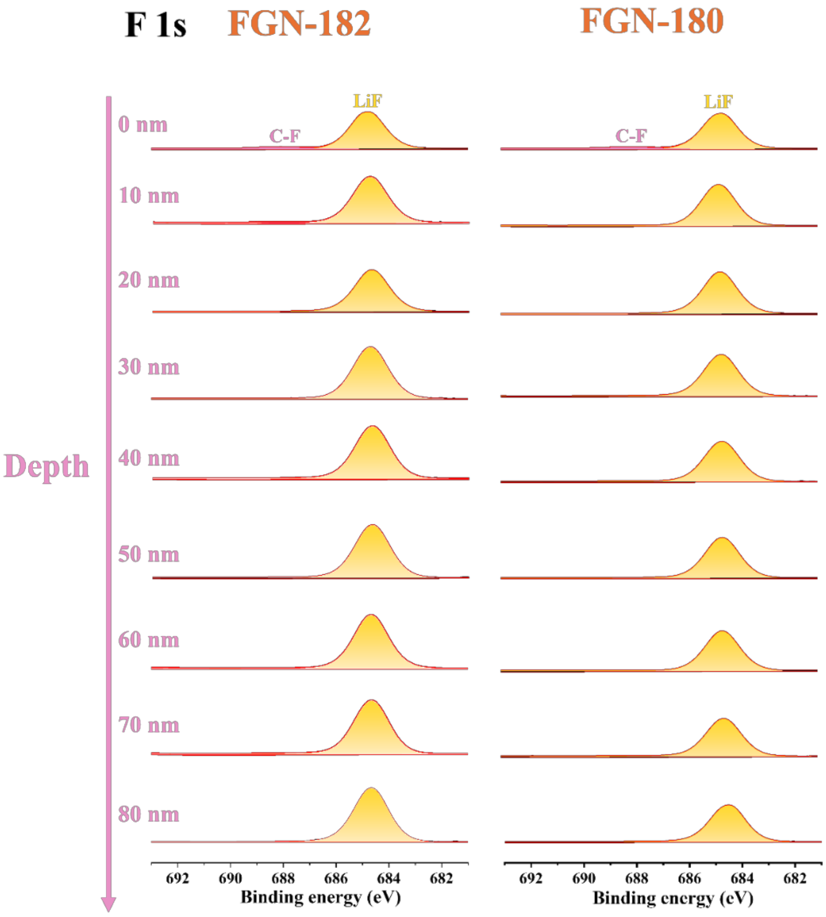

Fig. S19 The in-depth XPS spectra of SEI layer formed on Li metal cycled in FGN-182 and FGN-180 electrolytes of F 1s spectra

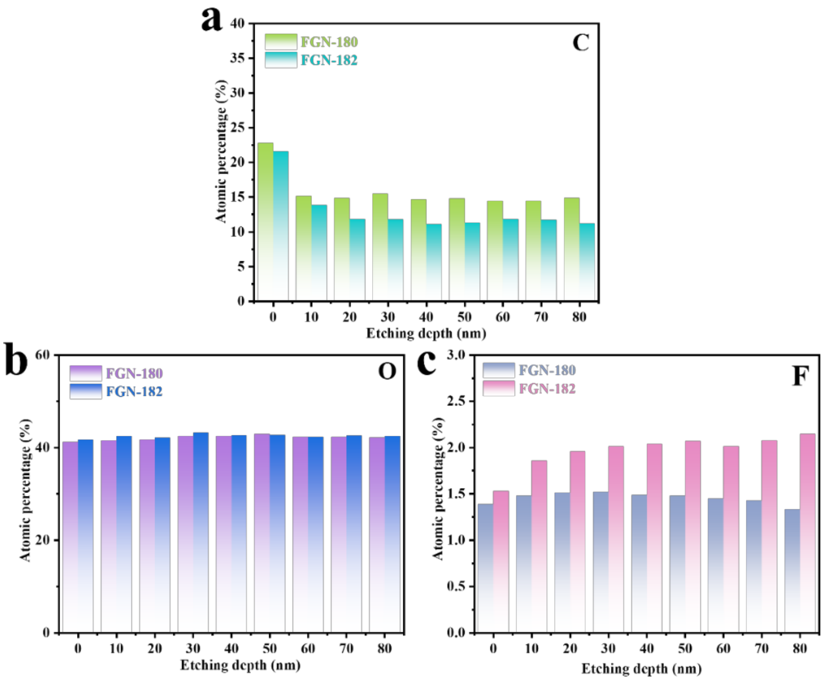

Fig. S20 Atomic percentage of SEI of (**a**) C, (**b**) O and (**c**) F for in FGN-182 and FGN-180 electrolytes with increasing etching depth

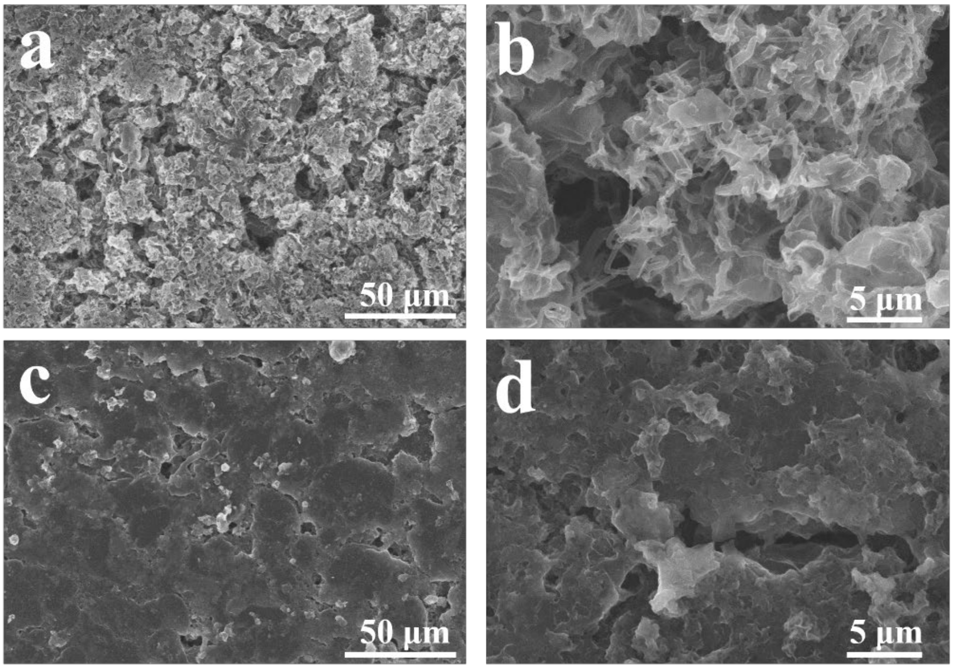

Fig. S21 Surface SEM images of symmetric cells after 50 cycles using (**a, b**) FGN-180 and (**c, d**) FGN-182 electrolytes

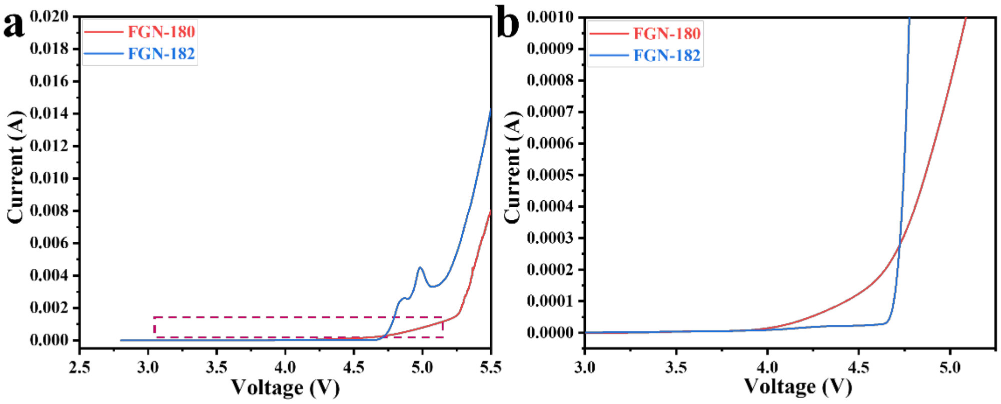

Fig. S22 (**a**) The linear sweep voltammetry curves of FGN-180 and FGN-182 electrolytes. (**b**) Partial enlarged drawing of (a)

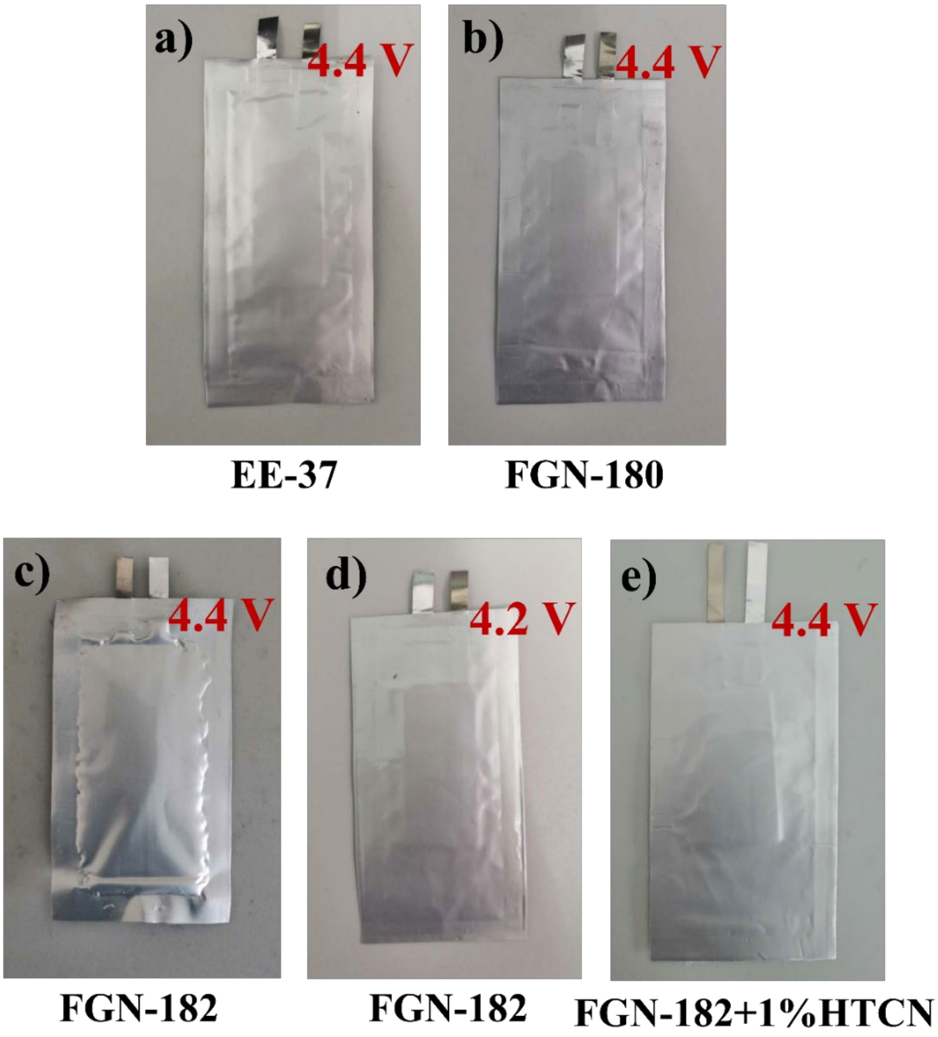

Fig. S23 The optical images of the pouch cells were taken after charging to different conditions (shown in the Fig.) using (**a**) EE37, (**b**) FGN-180, (**c**) FGN-182, (**d**) FGN-182 and (**e**) FGN-182+1%HTCN electrolytes

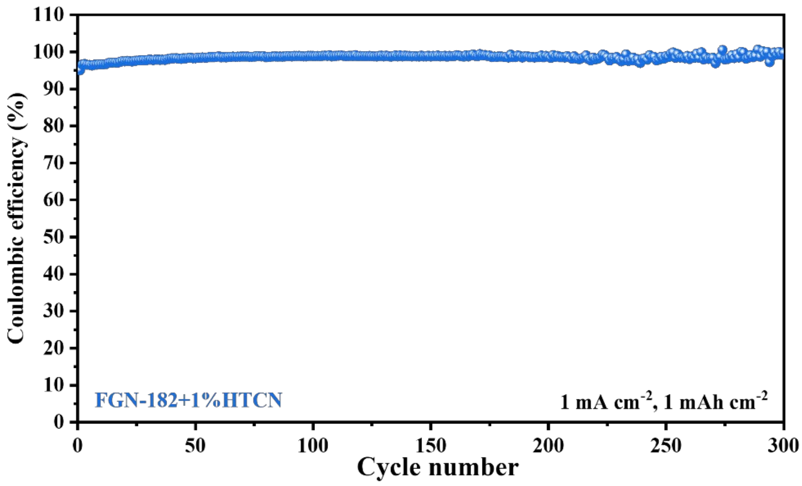

Fig. S24 CE of Li||Cu cells for FGN-182+1%HTCN electrolytes at 1 mA cm^{-2}, 1 mAh cm^{-2}

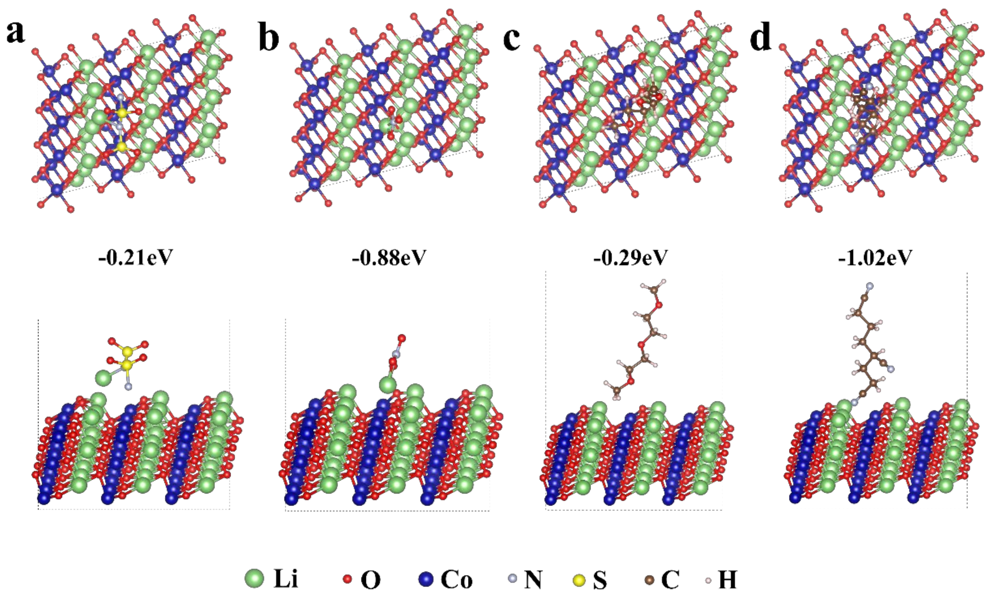

Fig. S25 Comparison of adsorption energies of LiFSI, $LiNO_3$, DEGDME, and HTCN on the (104) surface of $LiCoO_2$ from top and side views

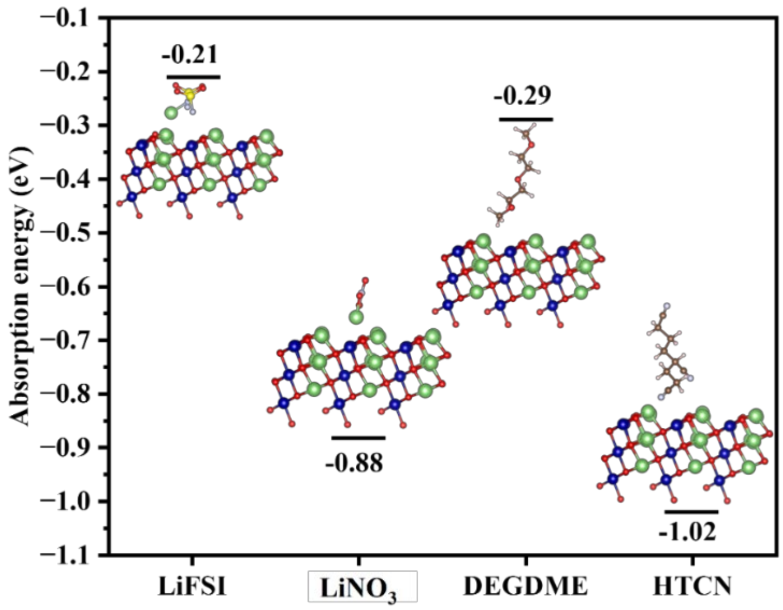

Fig. S26 Comparison of adsorption energies of LiFSI, $LiNO_3$, DEGDME, and HTCN on the (104) surface of $LiCoO_2$

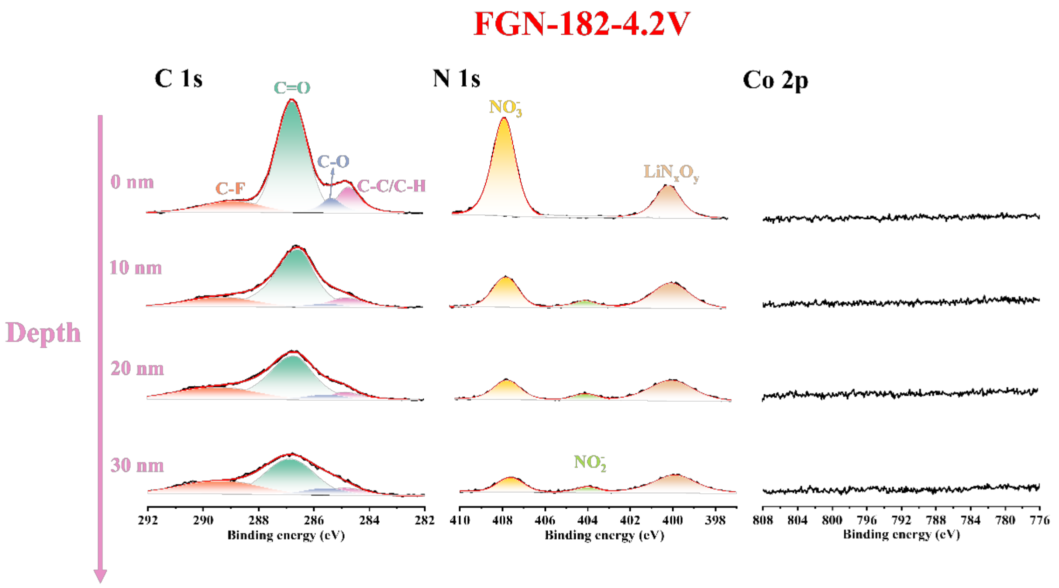

Fig. S27 The in-depth XPS spectra of the CEI film formed on LCO cycled in FGN-182 at a cut-off voltage range of 3−4.2 V

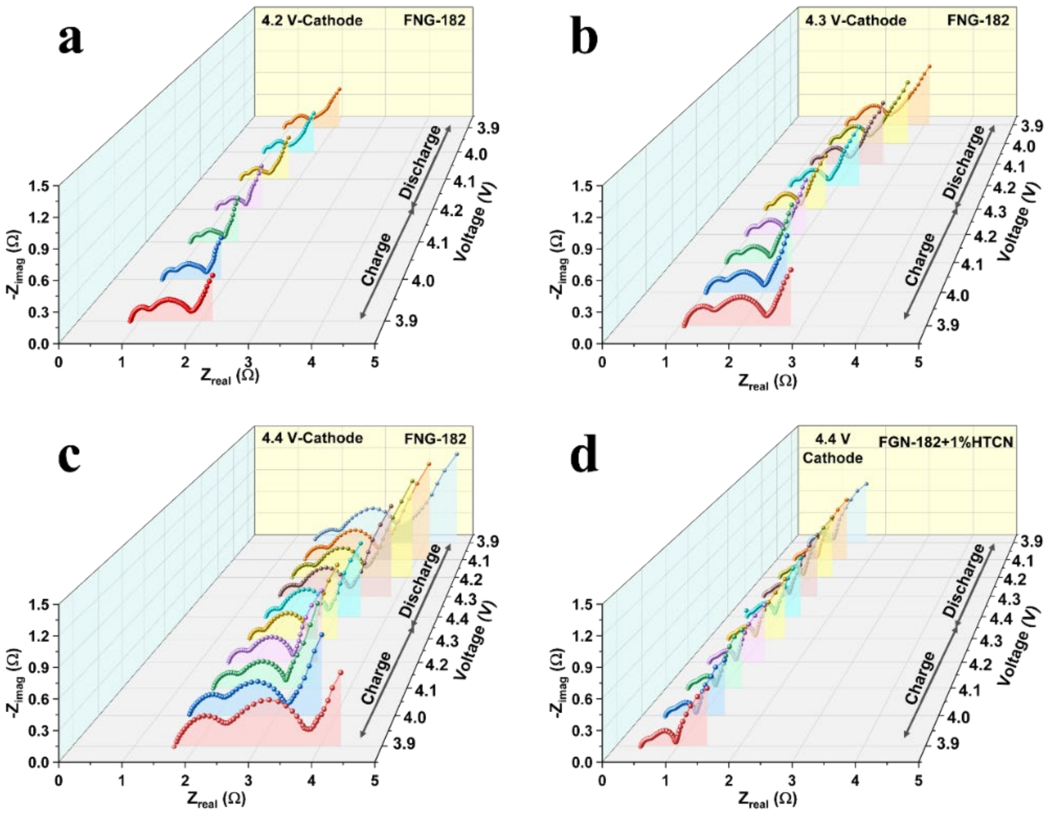

Fig. S28 The in-situ EIS profiles of LCO cathodes in LCO||Li full cells under different test conditions: **a-c**) FGN-182 and **d**) FGN-182+1%HTCN electrolytes

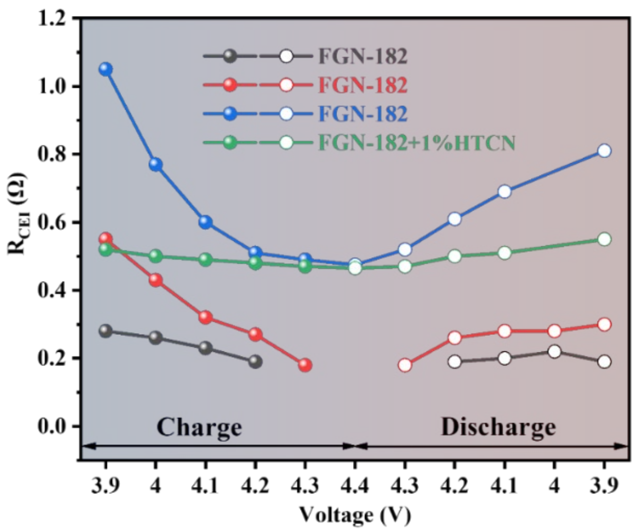

Fig. S29 The fitting results of R_{CEI} for LCO in LCO||Li full cells employing FGN-182 and FGN-182+1%HTCN electrolytes under different charge/discharge conditions

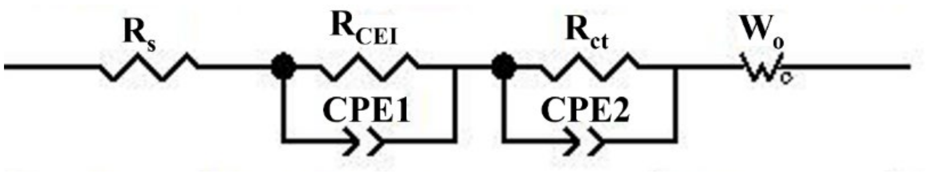

Fig. S30 The equivalent circuit model for the fitting of in-situ EIS profiles in Fig. S27

Table S1 Comparison of half-cell CE with reported electrolyte optimizations in the literature

Electrolyte formulation	Test method	Cycle life	Coulombic efficiency (Average)	Refs.
$LiFSI/DEGDME/LiNO_3$ (1:8:2)	Conventional Li||Cu cells	0.5 mA cm^{-2}, 1 mAh cm^{-2}, 1400 cycles	98.79%	**This work**
		1 mA cm^{-2}, 2 mAh cm^{-2}, 350 cycles	98.57%	
		1 mA cm^{-2}, 4 mAh cm^{-2}, 180 cycles	98.66%	
	Auerbach's method	0.5 mA cm^{-2}, 1 mAh cm^{-2}, 10 cycles	99.56%	
1.0 M $LiPF_6$ in EC/DEC (1:1) + 0.4 M $LiNO_3$ + 10 mM LiOTf	Auerbach's method	0.4 mA cm^{-2}, 0.8 mAh cm^{-2}, 10 cycles	98.5%	Energy Storage Mater. [S6]
0.25 M $LiNO_3$ + 0.9 M LiDFOB in DFNCA/FEC (1:1)	Conventional Li||Cu cells	0.5 mA cm^{-2}, 0.5 mAh cm^{-2}, 400 cycles	97%	ACS Nano [S7]
	Auerbach's method	0.5 mA cm^{-2}, 1 mAh cm^{-2}, 20 cycles	99%	
0.4 M $LiBF_4$ + 0.4 M LiTFSI	Auerbach's	0.5 mA cm^{-2}, 1 mAh cm^{-2},	99.85%	Nat. Energy [S8]

<table>
<tr><td>in THP + 20% FEC</td><td>method</td><td>10 cycles</td><td></td><td></td></tr>
<tr><td>1 M $LiPF_6$ in EC/DEC (1:1) + 2wt% TTFEB + 0.7wt%$LiNO_3$</td><td>Conventional Li||Cu cells</td><td>1 mA cm^{-2}, 1 mAh cm^{-2}, 270 cycles</td><td>98.6%</td><td>Nano Energy [S9]</td></tr>
<tr><td>2 M LiFSI in DME/BTFMD (1:3)</td><td>Conventional Li||Cu cells
Auerbach's method</td><td>1 mA cm^{-2}, 1 mAh cm^{-2}, 250 cycles
0.5 mA cm^{-2}, 1 mAh cm^{-2}, 10 cycles</td><td>99.17%
99.54%</td><td>ACS Energy Lett. [S10]</td></tr>
<tr><td>0.5 M $LiNO_3$ + 1 M LiDFOB in DME + 5%VC</td><td>Conventional Li||Cu cells</td><td>0.5 mA cm^{-2}, 0.5 mAh cm^{-2}, 320 cycles</td><td>97.6%</td><td>Adv. Funct. Mater. [S11]</td></tr>
<tr><td>0.3 M $LiNO_3$ + 1 M LiFSI in (PP13TFSI/DME) (1:4)</td><td>Conventional Li||Cu cells</td><td>2 mA cm^{-2}, 1 mAh cm^{-2}, 200 cycles</td><td>98%</td><td>Adv. Energy Mater. [S12]</td></tr>
<tr><td>1 M $LiPF_6$ in EC/DMC (1:1) + 0.5%$LiNO_3$ + 0.5%HFT</td><td>Auerbach's method</td><td>2 mA cm^{-2}, 1 mAh cm^{-2}, 10 cycles</td><td>97.1%</td><td>Adv. Energy Mater. [S13]</td></tr>
<tr><td>DFB/LiFSI/DMAC (0.75:1.00:0.50) + 5%EPC</td><td>Conventional Li||Cu cells
Auerbach's method</td><td>0.5 mA cm^{-2}, 0.5 mAh cm^{-2}, 260 cycles
0.5/1.5 mA cm^{-2}, 1 mAh cm^{-2}, 50 cycles</td><td>98.2%
98.7%</td><td>Angew Chem. Int. Ed. Engl. [S14]</td></tr>
<tr><td>0.1 M LiTFSI in FEC/FB (2:8)</td><td>Conventional Li||Cu cells
Auerbach's method</td><td>1 mA cm^{-2}, 1 mAh cm^{-2}, 100 cycles
0.5mA cm^{-2}, 1 mAh cm^{-2}, 10 cycles</td><td>98%
98.8%</td><td>Angew Chem. Int. Ed. Engl. [S15]</td></tr>
<tr><td>1.25 M $LiPF_6$ in asymF-DEC/FEC/VC (8:2:0.5)</td><td>Conventional Li||Cu cells
Auerbach's method</td><td>0.5 mA cm^{-2}, 1 mAh cm^{-2}, 250 cycles
0.5mA cm^{-2}, 1 mAh cm^{-2}, 10 cycles</td><td>98.73%
98.97%</td><td>Adv. Energy Mater. [S16]</td></tr>
<tr><td>2 M LiTFSI in DOL/THF/TTE (3:3:4)</td><td>Conventional Li||Cu cells
Auerbach's method</td><td>0.5 mA cm^{-2},1 mAh cm^{-2}, 250 cycles
0.5mA cm^{-2}, 1 mAh cm^{-2}, 10 cycles</td><td>99.12%
99.52%</td><td>Adv. Funct. Mater. [S17]</td></tr>
<tr><td>1 M $LiPF_6$ in EC/DMC (1:1) + 5%FEC + 0.025 M DIDP + 0.025 M TMSF + 0.1 M $LiNO_3$</td><td>Conventional Li||Cu cells
Auerbach's method</td><td>0.5 mA cm^{-2},1 mAh cm^{-2}, 130 cycles
0.5mA cm^{-2}, 1 mAh cm^{-2}, 20 cycles</td><td>98.3%
98.96%</td><td>Angew Chem. Int. Ed. Engl. [S18]</td></tr>
<tr><td>1 M $LiPF_6$ + 0.2 M $LiNO_3$ in EC/GTA/DMC (6:1:3)</td><td>Auerbach's method</td><td>0.5mA cm^{-2}, 1 mAh cm^{-2}, 10 cycles</td><td>99%</td><td>Angew Chem. Int. Ed. Engl. [S19]</td></tr>
<tr><td>1 M $LiPF_6$ in EC/DMC/FEC (10:10:1) + 0.1 M LiFSI + 0.1 M $LiNO_3$</td><td>Conventional Li||Cu cells</td><td>0.5mA cm^{-2}, 1 mAh cm^{-2}, 100 cycles</td><td>98.2%</td><td>Angew Chem. Int. Ed. Engl. [S20]</td></tr>
<tr><td>1 M LiFSI in EC/DMC (7:3) + 0.3 M $LiNO_3$ + 30 mM CTAB</td><td>Auerbach's method</td><td>0.5mA cm^{-2}, 1 mAh cm^{-2}, 10 cycles</td><td>99.4%</td><td>Nano-Micro Lett. [S21]</td></tr>
</table>

Table S2 Number and density of molecules in the simulation box

	**FGN-180**	**FGN-180.2**	**FGN-182**
LiFSI	100	100	100
DEGDME	800	800	800
$LiNO_3$	/	20	200

Table S3 The coordination number of Li^+

	FGN-180	FGN-180.2	FGN-182
S_{FSI} (within 3.2Å of Li)	2.069	1.849	0.899
F_{FSI} (within 4.1Å of Li)	2.273	2.059	1.042
O_{FSI} (within 1.9Å of Li)	2.071	1.852	0.903
O_{DEGDME} (within 1.9Å of Li)	2.778	2.370	1.587
O_{NO3} (within 1.9Å of Li)	/	0.496	2.037
N_{FSI} (within 4.2Å of Li)	2.062	1.866	0.901
N_{NO3} (within 3.0Å of Li)	/	0.481	1.99

Table S4 Comparison of full-cell cycling performance with reported electrolyte optimizations in the literature

**Electrolyte formulation**	**Electrolyte amount**	**Cell condition**	**Cycling condition & capacity retention**	**Refs.**
$LiFSI/DEGDME/LiNO_3$ (1:8:2)	≈20 g Ah^{-1}	Li (25 μm)||NMC111 (2.5 mAh cm^{-2}), N/P = 2.06 (coin cells)	3–4.2V, 0.5C cycling, 80%@140 cycles<br>3–4.2V, 1C cycling, 80%@165 cycles	**This work**
	5 g Ah^{-1}	Li (25 μm)||LCO (2.5 mAh cm^{-2}), N/P = 2 (pouch cells)	3–4.2V, 0.2C cycling, 80%@125 cycles	
		Li (25 μm)||LCO (2.8 mAh cm^{-2}), N/P = 1.8 (pouch cells)	3–4.3V, 0.2C cycling, 80%@122 cycles	
$LiFSI/DEGDME/LiNO_3$ (1:8:2) + 1%HTCN	5 g Ah^{-1}	Li (25 μm)||LCO (3 mAh cm^{-2}), N/P = 1.7 (pouch cells)	3–4.4V, 0.2C cycling, 80%@93 cycles	
1.0 M $LiPF_6$ in EC/DEC (1:1) + 0.4 M $LiNO_3$ + 10 mM LiOTf	50 μL<br>>90 g Ah^{-1}	Li (450 μm)||NMC811(2.0–3.0 mg cm^{-2}) N/P > 30 (coin cells)	3–4.3V, 2C cycling, 95.8%@250 cycles	Energy Storage Mater. [S6]
0.25 M $LiNO_3$ + 0.9M LiDFOB in DFNCA /FEC (1:1)	70 μL<br>≈51.8 g Ah^{-1}	Li (40 μm)||NMC811 (1.35 mAh cm^{-2}), N/P = 6.1 (coin cells)	3–4.3V, 0.25C cycling, 77%@100 cycles	ACS Nano [S7]
1 M $LiPF_6$ in EC/DEC (1:1) + 2wt% TTFEB + 0.7wt%$LiNO_3$	60 μL<br>≈14.8 g Ah^{-1}	Li (50 μm)||NMC811 (4.04 mAh cm^{-2}), N/P = 2.5 (coin cells)	2.8-4.4 V, 0.3C cycling, 81.5%@140 cycles	Nano Energy [S9]
2 M LiFSI in DME/BTFMD (1:3)	60 μL<br>≈28.5 g Ah^{-1}	Li (40 μm)||NMC811 (2.1 mAh cm^{-2}), N/P = 3.9 (coin cells)	3–4.3V, 1 C cycling, 82.2%@570 cycles	ACS Energy Lett. [S10]
	40 μL<br>≈20 g Ah^{-1}	Li (20 μm)||NMC811 (2.0 mAh cm^{-2}), N/P = 2.1 (coin cells)	3–4.4V, 1 C cycling, 80%@250 cycles	
0.5 M $LiNO_3$ + 1 M LiDFOB in DME + 5%VC	16.4 μL<br>≈9.8 g Ah^{-1}	Li (50 μm)||NMC811 (1.66 mAh cm^{-2}), N/P = 6.2 (coin cells)	3–4.4V, 0.33 C/0.66 C cycling, 62%@300 cycles	Adv. Funct. Mater. [S11]
0.3 M $LiNO_3$ + 1 M LiFSI in (PP13TFSI/DME) (1:4)	100 μL<br>≈138.8 g Ah^{-1}	Li (9 μm)||NMC811(0.72 mAh cm^{-2}), N/P = 2 (coin cells)	3–4.3 V, 2C/5C cycling, 69%@150 cycles	Adv. Energy Mater. [S12]
		Li (4.5 μm)||NMC811 (0.72 mAh cm^{-2}), N/P = 1 (coin cells)	3–4.3 V, 2C/5C cycling, 66%@100 cycles	

LiFSI:DMAC:DFB (0.75:1.00:0.50) + 5%EPC	50 μL ≈85.0 g Ah^{-1}	Li foil‖NMC811 (3 mg cm^{-2}), N/P >> 1 (coin cells)	2.8–4.3 V, 0.5 C cycling, 83.1%@300 cycles	Angew Chem. Int. Ed. Engl. [S14]
0.1 M LiTFSI in FEC/FB (2:8)	60 μL ≈88.2 g Ah^{-1}	Li (13.6 μm)‖$LiFePO_4$ (0.68 mAh cm^{-2}), N/P = 4 (coin cells)	2.5–3.9 V, 0.3 C cycling, 83.3%@160 cycles	Angew Chem. Int. Ed. Engl. [S15]
	60 μL ≈70.5 g Ah^{-1}	Li (12.8 μm)‖NMC811 (0.85 mAh cm^{-2}), N/P = 3 (coin cells)	3–4.3 V, 0.3 C cycling, 85.9%@150 cycles	
1.25 M LiPF6 in asymF-DEC/FEC/VC (8:2:0.5)	70 μL ≈18 g Ah^{-1}	Li (50 μm)‖NMC811 (3.82 mAh cm^{-2}), N/P = 2.62 (coin cells)	2.9–4.3 V, 0.4C cycling, 80%@240 cycles	Adv. Energy Mater. [S16]
2 M LiTFSI in DOL/THF/TTE (3:3:4)	75 μL ≈51.0 g Ah^{-1}	Li (22 μm)‖NMC811 (1.47 mAh cm^{-2}), N/P=3 (coin cells)	2.8–4.3 V, 0.5C cycling, 85.2%@100 cycles	Adv. Funct. Mater. [S17]
1 M $LiPF_6$ in EC/DMC (1:1) + 5%FEC + 0.025 M DIDP + 0.025 M TMSF + 0.1 M $LiNO_3$	50 μL ≈29.0 g Ah^{-1}	Li (400 μm)‖NMC622 (1.72 mAh cm^{-2}), N/P=48 (coin cells)	2.9–4.3 V, 1C cycling, 82.2%@250 cycles<br>2.9–4.3 V, 1C cycling, 81.9%@150 cycles	Angew Chem. Int. Ed. Engl. [S18]
1 M $LiPF_6$ + 0.2 M $LiNO_3$ in EC/GTA/DMC (6:1:3)	70 μL ≈35 g Ah^{-1}	Li (50 μm)‖NMC811 (2 mAh cm^{-2}), N/P=5.15 (coin cells)	2.8–4.3 V, 0.5C cycling, 84.2%@180 cycles	Angew Chem. Int. Ed. Engl. [S19]
	70 μL ≈19.4 g Ah^{-1}	Li (50 μm)‖NMC811 (3.6 mAh cm^{-2}), N/P=2.86 (coin cells)	2.8–4.5 V, 0.5C cycling, 90.2%@80 cycles	
1 M $LiPF_6$ in EC/DMC/FEC (10:10:1) + 0.1 M LiFSI + 0.1 M $LiNO_3$	50 μL ≈20.8 g Ah^{-1}	Li (450 μm)‖NMC523 (12 mg cm^{-2}), N/P=44 (coin cells)	2.9–4.3 V, 1C cycling, 80.2%@300 cycles	Angew Chem. Int. Ed. Engl. [S20]
1 M LiFSI, 0.3 M $LiNO_3$, 30 mM CTAB in EC/DMC (7:3)	17.6 μL ≈11.1 g Ah^{-1}	Li (95 μm)‖$LiFePO_4$ (1.58 mAh cm^{-2}), N/P=12 (coin cells)	2.5–3.8 V, 0.5C cycling, 80%@180 cycles	Nano-Micro Lett. [S21]

The calculation of electrolyte content is based on the assumption of a density of 1 g/mL in each electrolyte

Supplementary References

[S1] J. Evans, C. A. Vincent, and P. G. Bruce, Electrochemical measurement of transference numbers in polymer electrolytes. Polymer **28**, 2324-2328 (1987). https://doi.org/10.1016/0032-3861(87)90394-6

[S2] P. Giannozzi, S. Baroni, N. Bonini, M. Calandra, R. Car et al., QUANTUM ESPRESSO: a modular and open-source software project for quantum simulations of materials. J. Condens. Matter Phys. **21**, 395502 (2009). https://doi.org/10.1088/0953-8984/21/39/395502

[S3] D. Van Der Spoel, E. Lindahl, B. Hess, G. Groenhof, A. E. Mark et al., GROMACS: fast, flexible, and free. J. Comput. Chem. **26**, 1701-1718 (2005). https://doi.org/10.1002/jcc.20291

[S4] M. J. Robertson, J. Tirado-Rives, and W. L. Jorgensen, Improved peptide and protein torsional energetics with the OPLS-AA force field. J. Chem. Theory Comput. **11**, 3499-3509 (2015). https://doi.org/10.1021/acs.jctc.5b00356

[S5] L. Martínez, R. Andrade, E. G. Birgin, and J. M. PACKMOL, A package for building initial configurations for molecular dynamics simulations, J. Comput. Chem. **30**, 2157-2164 (2009). https://doi.org/10.1002/jcc.21224

[S6] D. Chai, Y. Zhu, C. Guan, T. Zhang, S. Tang et al., Achieving stable interphases toward lithium metal batteries by a dilute and anion-rich electrolyte. Energy Storage Mater. **62**, 102957 (2023). https://doi.org/10.1016/j.ensm.2023.102957

[S7] W. H. Hou, P. Zhou, H. Gu, Y. Ou, Y. Xia, et al., Fluorinated carbamate-based electrolyte enables anion-dominated solid electrolyte interphase for highly reversible Li metal anode. ACS Nano. **17**, 17527-17535 (2023). https://doi.org/10.1021/acsnano.3c06088

[S8] H. Kwon, H. Kim, J. Hwang, W. Oh, Y. Roh, et al., Borate–pyran lean electrolyte-based Li-metal batteries with minimal Li corrosion. Nat. Energy **9**, 57-69 (2023). https://doi.org/10.1038/s41560-023-01405-6

[S9] K. Wang, X. Wang, W. Ahmad, J. Zhao, H. Li, et al., Liang. Synergistic dual-additive regulated carbonate electrolyte stabilizes bidirectional interface for aggressive Ni-rich Li-metal full batteries. Nano Energy **128**, 109862 (2024). https://doi.org/10.1016/j.nanoen.2024.109862

[S10] K. Lee, S.-H. Kwon, J. Kim, E. Park, I. Kim, et al., Fluorinated cyclic ether diluent for high-voltage lithium metal batteries. ACS Energy Lett. **9**, 2201-2211 (2024). https://doi.org/10.1021/acsenergylett.4c00481

[S11] Z. Jiang, T. Yang, C. Li, J. Zou, H. Yang et al., Synergistic additives enabling stable cycling of ether electrolyte in 4.4 V Ni-rich/Li metal batteries. Adv. Funct. Mater. **33**, 2306868 (2023). https://doi.org/10.1002/adfm.202306868

[S12] K. Ding, E. J. Begin, S. Yuan, M. Zhong, Y. Wang et al., Boosting fast-charging capability of high-voltage Li metal batteries with ionic liquid modified ethereal electrolyte. Adv. Energy Mater. **13**, 2302443 (2023). https://doi.org/10.1002/aenm.202302443

[S13] J. Jiang, M. Li, X. Liu, J. Yi, Y. Jiang, et al., Multifunctional additives to realize dendrite-free lithium deposition in carbonate electrolytes toward low-temperature Li metal batteries. Adv. Energy Mater. 2400365 (2024). https://doi.org/10.1002/aenm.202400365

[S14] R. He, K. Deng, D. Mo, X. Guan, Y. Hu et al., Active diluent-anion synergy strategy regulating nonflammable electrolytes for high-efficiency Li metal batteries. Angew Chem. Int. Ed. Engl. **63**, e202317176 (2024). https://doi.org/10.1002/anie.202317176

[S15] P. Li, Z. Zhang, Z. Zhao, X. Zhang, H. Zhang et al., Localized medium concentration electrolyte with fast kinetics for lithium metal batteries. Angew Chem. Int. Ed. **63**, e202319090 (2024). https://doi.org/10.1002/anie.202319090

[S16] L. Deng, L. Dong, Z. Wang, Y. Liu, J. Zhan et al., Asymmetrically-fluorinated electrolyte molecule design for simultaneous achieving good solvation and high inertness to enable stable lithium metal batteries. Adv. Energy Mater. **14**, 2303652 (2023). https://doi.org/10.1002/aenm.202303652

[S17] R. Gu, D. Zhang, S. Zhu, J. Xu, K. Ding et al., An ether-based electrolyte solvation strategy for long-term stability and ultra-low temperature Li-metal batteries. Adv. Funct. Mater. **34**, 2310747 (2024). https://doi.org/10.1002/adfm.202310747

[S18] Y. Gao, G. Wu, W. Fang, Z. Qin, T. Zhang et al., Transesterification induced multifunctional additives enable high-performance lithium metal batteries. Angew Chem. Int. Ed. Engl. **63**, e202403668 (2024). https://doi.org/10.1002/anie.202403668

[S19] Z. Jin, Y. Liu, H. Xu, T. Chen, and C. Wang, Intrinsic solubilization of lithium nitrate in ester electrolyte by multivalent low-entropy-penalty design for stable lithium-metal batteries. Angew Chem. Int. Ed. Engl. **63**, e202318197 (2024). https://doi.org/10.1002/anie.202318197

[S20] Z. Wen, W. Fang, F. Wang, H. Kang, S. Zhao et al., Dual-salt electrolyte additive enables high moisture tolerance and favorable electric double layer for lithium metal battery. Angew Chem. Int. Ed. Engl. **63**, e202314876 (2024). https://doi.org/10.1002/anie.202314876

[S21] Z. Sun, J. Yang, H. Xu, C. Jiang, Y. Niu et al., Enabling an inorganic-rich interface via cationic surfactant for high-performance lithium metal batteries. Nano-Micro Lett. **16**, 141 (2024). https://doi.org/10.1007/s40820-024-01364-x
